# Safety and immunogenicity of the ChAdOx1 nCoV-19 (AZD1222) vaccine against SARS-CoV-2 in people living with and without HIV in South Africa: an interim analysis of a randomised, double-blind, placebo-controlled, phase 1B/2A trial

**DOI:** 10.1016/S2352-3018(21)00157-0

**Published:** 2021-08-17

**Authors:** Shabir A Madhi, Anthonet L Koen, Alane Izu, Lee Fairlie, Clare L Cutland, Vicky Baillie, Sherman D Padayachee, Keertan Dheda, Shaun L Barnabas, Qasim Ebrahim Bhorat, Carmen Briner, Parvinder K Aley, Sutika Bhikha, Tandile Hermanus, Elizea Horne, Aylin Jose, Prudence Kgagudi, Teresa Lambe, Masebole Masenya, Mduduzi Masilela, Nonhlanhla Mkhize, Andrew Moultrie, Christian K Mukendi, Thandeka Moyo-Gwete, Amit J Nana, Ayanda Nzimande, Faeezah Patel, Sarah Rhead, Carol Taoushanis, Asha Thombrayil, Samuel van Eck, Merryn Voysey, Tonya L Villafana, Johan Vekemans, Sarah C Gilbert, Andrew J Pollard, Penny L Moore, Gaurav Kwatra, Wits VIDA COVID team

**Affiliations:** a South African Medical Research Council Vaccines and Infectious Diseases Analytics Research Unit, Faculty of Health Sciences, University of the Witwatersrand, Johannesburg, South Africa; b Department of Science and Innovation/National Research Foundation South African Research Chair Initiative in Vaccine Preventable Diseases Unit, Faculty of Health Sciences, University of the Witwatersrand, Johannesburg, South Africa; c African Leadership in Vaccinology Expertise, Faculty of Health Sciences, University of the Witwatersrand, Johannesburg, South Africa; d Wits Reproductive Health and HIV Institute, Faculty of Health Sciences, University of the Witwatersrand, Johannesburg, South Africa; e Perinatal HIV Research Unit, Faculty of Health Sciences, University of the Witwatersrand, Johannesburg, South Africa; f SAMRC Antibody Immunity Research Unit, School of Pathology, Faculty of Health Sciences, University of the Witwatersrand, Johannesburg, South Africa; g Setshaba Research Centre, Tshwane, South Africa; h Division of Pulmonology, Groote Schuur Hospital and the University of Cape Town, Cape Town, South Africa; i Faculty of Infectious and Tropical Diseases, Department of Immunology and Infection, London School of Hygiene & Tropical Medicine, London, UK; j Family Centre for Research With Ubuntu, Department of Paediatrics, University of Stellenbosch, Cape Town, South Africa; k Soweto Clinical Trials Centre, Soweto, South Africa; l Oxford Vaccine Group, University of Oxford, Oxford, UK; m Department of Paediatrics, University of Oxford, Oxford, UK; n NIHR Oxford Biomedical Research Centre, Oxford, UK; o National Institute for Communicable Diseases Division of the National Health Laboratory Service, Johannesburg, South Africa; p Jenner Institute, Nuffield Department of Medicine, University of Oxford, UK; q AstraZeneca Biopharmaceuticals, Cambridge, UK

## Abstract

**Background:**

People living with HIV are at an increased risk of fatal outcome when admitted to hospital for severe COVID-19 compared with HIV-negative individuals. We aimed to assess safety and immunogenicity of the ChAdOx1 nCoV-19 (AZD1222) vaccine in people with HIV and HIV-negative individuals in South Africa.

**Methods:**

In this ongoing, double-blind, placebo-controlled, phase 1B/2A trial (COV005), people with HIV and HIV-negative participants aged 18–65 years were enrolled at seven South African locations and were randomly allocated (1:1) with full allocation concealment to receive a prime-boost regimen of ChAdOx1 nCoV-19, with two doses given 28 days apart. Eligibility criteria for people with HIV included being on antiretroviral therapy for at least 3 months, with a plasma HIV viral load of less than 1000 copies per mL. In this interim analysis, safety and reactogenicity was assessed in all individuals who received at least one dose of ChAdOx1 nCov 19 between enrolment and Jan 15, 2021. Primary immunogenicity analyses included participants who received two doses of trial intervention and were SARS-CoV-2 seronegative at baseline. This trial is registered with ClinicalTrials.gov, NCT04444674, and the Pan African Clinicals Trials Registry, PACTR202006922165132.

**Findings:**

Between June 24 and Nov 12, 2020, 104 people with HIV and 70 HIV-negative individuals were enrolled. 102 people with HIV (52 vaccine; 50 placebo) and 56 HIV-negative participants (28 vaccine; 28 placebo) received the priming dose, 100 people with HIV (51 vaccine; 49 placebo) and 46 HIV-negative participants (24 vaccine; 22 placebo) received two doses (priming and booster). In participants seronegative for SARS-CoV-2 at baseline, there were 164 adverse events in those with HIV (86 vaccine; 78 placebo) and 237 in HIV-negative participants (95 vaccine; 142 placebo). Of seven serious adverse events, one severe fever in a HIV-negative participant was definitely related to trial intervention and one severely elevated alanine aminotranferase in a participant with HIV was unlikely related; five others were deemed unrelated. One person with HIV died (unlikely related). People with HIV and HIV-negative participants showed vaccine-induced serum IgG responses against wild-type Wuhan-1 Asp614Gly (also known as D614G). For participants seronegative for SARS-CoV-2 antigens at baseline, full-length spike geometric mean concentration (GMC) at day 28 was 163·7 binding antibody units (BAU)/mL (95% CI 89·9–298·1) for people with HIV (n=36) and 112·3 BAU/mL (61·7–204·4) for HIV-negative participants (n=23), with a rising day 42 GMC booster response in both groups. Baseline SARS-CoV-2 seropositive people with HIV demonstrated higher antibody responses after each vaccine dose than did people with HIV who were seronegative at baseline. High-level binding antibody cross-reactivity for the full-length spike and receptor-binding domain of the beta variant (B.1.351) was seen regardless of HIV status. In people with HIV who developed high titre responses, predominantly those who were receptor-binding domain seropositive at enrolment, neutralising activity against beta was retained.

**Interpretation:**

ChAdOx1 nCoV-19 was well tolerated, showing favourable safety and immunogenicity in people with HIV, including heightened immunogenicity in SARS-CoV-2 baseline-seropositive participants. People with HIV showed cross-reactive binding antibodies to the beta variant and Asp614Gly wild-type, and high responders retained neutralisation against beta.

**Funding:**

The Bill & Melinda Gates Foundation, South African Medical Research Council, UK Research and Innovation, UK National Institute for Health Research, and the South African Medical Research Council.

## Introduction

Vaccines against SARS-CoV-2 are being deployed globally to prevent COVID-19. Included among the first-generation COVID-19 vaccines authorised for emergency use is ChAdOx1 nCoV-19 (AZD1222), a non-replicating chimpanzee adenovirus-vectored vaccine that expresses the full-length spike (FLS) glycoprotein gene of SARS-CoV-2.^1^, ^2^ Safety and efficacy of ChAdOx1 nCoV-19 in clinical trials have been demonstrated,^3^ and immunogenicity studies have shown that ChAdOx1 nCoV-19 induces antibody responses specific to the spike protein, and to the receptor-binding domain (RBD) of the spike protein 28 days after the first dose in all adults.^4^

The SARS-CoV-2 spike genome has accumulated mutations resulting in the emergence of variants, including the beta (B.1.351) lineage first identified in South Africa.^5^ There are few published reports on the safety and immunogenicity of ChAdOx1 nCoV-19 and other COVID-19 vaccines in people with HIV, and even fewer reports specific to people living in Africa.^6^ Compared with HIV-negative individuals, people with HIV are at greater risk for infectious diseases, such as influenza, including during antiretroviral therapy (ART),^7^ and are at higher risk of a fatal outcome when admitted to hospital for severe COVID-19.^8^ Risk factors for severe COVID-19 in people with HIV include more advanced stage of HIV/AIDS, the HIV-1 infection not being virally suppressed, and CD4 counts below 500 cells per μL.

The US Centers for Disease Control and Prevention advises that people with HIV can choose to be vaccinated against COVID-19, but might have reduced immune responses to the vaccine,^9^ whereas WHO recommends that people with HIV should be immunised with COVID-19 vaccines.^10^, ^11^ Although there are an estimated 38 million people with HIV globally, there is limited knowledge on the safety and immunogenicity of COVID-19 vaccines in this population.^12^, ^13^ This disparity is particularly pertinent to sub-Saharan Africa, where more than 80% of the global population of people with HIV live, including 7·5 million in South Africa.^13^

Here, we report interim results from a multicentre, randomised, double-blind, placebo-controlled, phase 1B/2A trial (COV005) assessing the safety and immunogenicity of ChAdOx1 nCoV-19 in people with and without HIV-1 in South Africa.

## Methods

### Study design

This interim analysis of the ongoing, adaptive, randomised, double-blind, placebo-controlled, phase 1B/2A trial (COV005) assessed the safety and immunogenicity of the ChAdOx1 nCoV-19 vaccine in South Africa. Study details were published previously.^6^ Briefly, trial enrolment began on June 24, 2020, and is ongoing in seven South African sites (research centres, hospitals, and clinical trials centre) in accordance with the principles of the Declaration of Helsinki and Good Clinical Practice Guidelines.^14^ The COV005 study was approved by the South African Health Products Regulatory Authority and the ethics committees of the University of Oxford, University of the Witwatersrand, Stellenbosch University, and University of Cape Town. The trial protocol (version 6.0) is available online.^15^

**Research in context**

**Evidence before this study**

We searched PubMed for peer-reviewed articles published between Jan 1, 2019, and June 29, 2021, with no language restrictions, using the terms “Safety” AND “COVID-19” AND “vaccine” AND “HIV”. Our search returned one peer-reviewed study that explored safety and immunogenicity of the ChAdOx1 nCoV-19 (AZD1222) vaccine in people with HIV in the UK, findings of which deemed the vaccine to be safe and immunogenic in people with HIV that are well controlled on antiretroviral therapy. We did not find any reports that evaluated safety and immunogenicity of COVID-19 vaccines in this population in Africa.

**Added value of this study**

This interim analysis of the COV005 study provides novel evidence of the safety and immunogenicity of the ChAdOx1 nCoV-19 vaccine in people with and without HIV in South Africa. Because people with HIV are at greater risk for infectious diseases and are at higher risk of a fatal outcome when admitted to hospital for severe COVID-19 than are the general population, our results provide reassurance of protection following ChAdOx1 nCoV-19 vaccination in this population. Furthermore, our results show that high neutralising antibody titres in people with HIV who were seropositive for SARS-CoV-2 at baseline were associated with a preserved ability to neutralise the beta (B.1.351) variant of concern, though at reduced titres; which was not evident in vaccinees without previous SARS-CoV-2 infection. This finding is of particular value in areas with high burdens of previous SARS-CoV-2 infection.

**Implications of all the available evidence**

These interim findings are vital for informing the clinical management of people with HIV during the COVID-19 pandemic. Our analysis suggests that priming by natural infection with the ancestral virus Asp614Gly wild-type before vaccination with ChAdOx1 nCoV-19 could lead to heightened neutralising antibody titres with relative preservation of activity against the beta variant compared with those who were SARS-CoV-2 seronegative when receiving the first vaccine dose.

### Participants

Volunteers from the general community were invited to participate in the study by way of public announcement and advertising on social media. Eligible participants were healthy adults aged 18–65 years with and without HIV in South Africa. HIV status was tested before enrolment. As this was a phase 1B/2A trial, inclusion criteria for people with HIV were restrictive, including those on stable ART for at least 3 months and required an HIV-1 viral load of less than 1000 copies per mL within 2 weeks of randomisation. All participants were required to test seronegative for hepatitis B surface antigen. Participants with abnormalities (grade ≥2) in full blood count, urea, and electrolyte tests, or liver function tests were excluded, according to the Division of AIDS Grading Criteria (version 2.1, July 2017). Full exclusion and inclusion criteria are provided in the trial protocol.^15^ All participants were fully informed about trial procedures and possible risks before giving written informed consent.

### Randomisation and masking

Details of randomisation have been previously published.^14^ Briefly, people with HIV and HIV-negative participants were randomly assigned (1:1), via a computer-generated system, with full allocation concealment, to receive two intramuscular injections of either ChAdOx1 nCoV-19 (vaccine group) or saline placebo (0·9% sodium chloride; placebo group), given 28 days apart.

### Clinical procedures

As previously described,^6^ between June 24, 2020, and July 29, 2020, we enrolled a cohort of 70 HIV-negative individuals (group 1) for intensive safety and immunogenicity monitoring, followed by wider enrolment of HIV-negative individuals (group 2) for further safety, immunogenicity, and efficacy assessment. Group 2 data are described elsewhere.^15^ Between Aug 17, 2020, and Nov 12, 2020, we enrolled people with HIV (group 3) for intensive safety and immunogenicity assessment. This interim analysis compares data obtained from group 1 (HIV-negative individuals) and group 3 (people with HIV), which both involved intensive immunogenicity monitoring.

The original protocol (version 1.0) included a nasal swab to test for SARS-CoV-2 infection at the time of randomisation, irrespective of symptomatology, and blood samples taken for serology analysis of SARS-CoV-2 infection. After 24 participants were enrolled in group 1, seven (29%) participants had positive PCR tests for SARS-CoV-2 on nasal swabs collected on the day of randomisation. The protocol was, therefore, amended to include screening for SARS-CoV-2 via PCR within 96 h of randomisation, and a positive SARS-CoV-2 PCR test was added as an exclusion criterion, implemented before enrolment of people with HIV began.

For group 1, the sample size was increased from 50 to 70 to replace individuals who tested positive for SARS-CoV-2 infection at randomisation during the initial phase of enrolment. For group 3, the sample size was increased from 50 to 100 owing to a high (35–45%) level of SARS-CoV-2 infection opportunistically identified in residual blood samples from people with HIV in South Africa as the first wave was subsiding.^16^ We estimated that the increase in sample size would result in about 50 individuals in group 1 and 70 in group 3 who would remain seronegative for COVID-19 until 2 weeks after the booster dose and, therefore, evaluable for the main immunogenicity analysis that was restricted to those who were SARS-CoV-2 naive at enrolment.

ChAdOx1 nCoV-19 is formulated at 5 × 10^10^ virus particles per dose. In this trial, three different batches of ChAdOx1 nCoV-19 were manufactured and used as detailed in the appendix (pp 1–2). We used PCR to test for SARS-CoV-2 infection at baseline and throughout the trial as previously described.^15^

### Laboratory procedures

Expression plasmids encoding SARS-CoV-2 FLS and RBD were obtained from Florian Krammer (Mount Sinai, NY, USA). The recombinant FLS and RBD proteins from either the Asp614Gly (also known as D614G) wild-type or beta SARS-CoV-2 variant were expressed and purified as described previously^17^ and were coupled to magnetic microspheres (Bio-Rad, Philadelphia, PA, USA) using a two-step carbodiimide reaction.^18^

We analysed immunogenicity on day 0, before administration of the first placebo or vaccine dose (priming dose), on day 28 after the priming dose, the day that the second dose of placebo or vaccine (booster dose) was administered, and on day 42 (14 days after the booster). Singleplex bead-based immunoassays were developed on the Luminex platform to quantitatively measure serum IgG binding to FLS and RBD. We developed an in-house reference serum by pooling convalescent serum from adults with COVID-19. This interim reference serum was calibrated against a research reagent for anti-SARS-CoV-2 antibody (code 20/130 supplied by National Institute for Biological Standards and Control, Herts, UK). This research reagent for SARS-CoV-2 RNA was used for the development and evaluation of serological assays for the detection of antibodies against SARS-CoV-2. Details of the binding antibody units (BAU) for viral components can be found online. The BAU assigned to the in-house reference serum were 1242 BAU/mL for RBD IgG and 2819 BAU/mL for FLS IgG. Luminex assay sensitivity and specificity are described in the appendix (p 1).

Humoral responses at baseline and after vaccination were assessed with ELISA-based antibody binding assays to recombinant SARS-CoV-2 RBD and FLS proteins from Asp614Gly wild-type and the beta variant as previously described.^18^ Additional details are given in the appendix (p 1).

To determine serum concentrations of SARS-CoV-2 neutralising antibodies, we used two pseudovirus neutralisation assays. Samples from HIV-negative participants were tested using a SARS-CoV-2 Asp614Gly wild-type neutralisation assay at the National Institute for Communicable Diseases (South Africa) using pseudoviruses produced by cotransfection with a lentiviral backbone (HIV-1 pNL4.luc) as previously described.^17^ For samples from people with HIV, which might be affected by antiretroviral drugs, samples were first screened for antiretroviral-mediated background activity using murine leukaemia virus (MLV) envelope pseudotyped viruses in an MLV backbone.^19^ Of 51 samples, 18 (35%) showing background presumed to be mediated by antiretrovirals were excluded from further testing; acceptable samples were tested using a SARS-CoV-2-specific microneutralisation assay in the same MLV backbone expressing Asp614Gly wild-type. Comparison of the two assays on 56 samples showed high levels of concordance (*r*=0·8561, p<0·0001) for Asp614Gly wild-type-mediated neutralisation between these two assays (appendix p 31). We used a subset of 40 samples, which were shown to be seropositive for Asp614Gly wild-type FLS at day 42, to evaluate neutralisation against the beta variant. IgG was purified from 100 μL of plasma using protein A, and the eluate was normalised back to input volumes. IgG was tested against both Asp614Gly wild-type and the beta variant. High levels of concordance (*r*=0·9165, p=0·0054) between plasma-mediated and IgG-mediated neutralisation of Asp614Gly wild-type was seen in 23 samples (appendix p 32).

### Outcomes

The primary endpoint in both HIV-negative participants and people with HIV was the safety, tolerability, and reactogenicity profile of ChAdOx1 nCoV-19. Tolerability was assessed by local and systemic reactogenicity and adverse events, stratified by SARS-CoV-2 serostatus at enrolment. In people with HIV, the coprimary endpoint was cellular and humoral immunogenicity of ChAdOx1 nCoV-19, as assessed by quantification of serum antibody (IgG) to SARS-CoV-2 FLS protein, RBD, and virus neutralising antibody assays against pseudotyped SARS-CoV-2 virus, stratified by SARS-CoV-2 serostatus at enrolment. The coprimary endpoint of efficacy of ChAdOx1 nCoV-19 against COVID-19 in HIV-negative participants is reported elsewhere.^6^

Coprimary endpoints and unsolicited adverse events were assessed at days 7 and 28 after vaccination, respectively.

Baseline assessments included review of inclusion and exclusion criteria, medical history, vital sign measurements, history-directed clinical examination, and collection of serum for SARS-CoV-2 serology. All participants had SARS-CoV-2 PCR testing within 96 h of randomisation, and on days 7, 14, 28, 35, 42, and 56 after randomisation. Baseline SARS-CoV-2 serostatus was determined by testing participant serum with a colorimetric, plate-based RBD IgG ELISA assay, as previously described.^17^ Participants were also reminded to contact the trial site if they experienced specific symptoms associated with COVID-19.

In this interim analysis, we report solicited adverse events (reactogenicity) within 7 days of each vaccine or placebo dose, and unsolicited adverse events during the study period (28 days). Safety evaluations included analysis of full blood count, urine, and electrolytes, and liver function tests, which were all assessed before randomisation, on days 3 and 7 after randomisation, on the day the booster dose was given, and on days 14 and 28 after the booster dose. Follow-up will continue for 12 months after enrolment. This interim analysis includes adverse event data reported by Jan 15, 2021 (inclusive).

### Statistical analysis

The safety analysis set, used for safety and reactogenicity analyses, included all randomised individuals who received at least one dose of trial intervention between enrolment and Jan 15, 2021. Primary immunogenicity analyses included participants who received two doses of trial intervention and were SARS-CoV-2 seronegative at baseline, with further analyses done in those who were RBD seropositive at baseline. Immunogenicity analyses were restricted to individuals who were not SARS-CoV-2 seropositive, with seropositive individuals included up until the time of infection at any time between day 0 and day 42. Summary statistics for demographic and clinical characteristics are reported as medians (IQR) for quantitative measurements and as counts and proportions for categorical variables. Antibody concentrations or titres are summarised as geometric mean concentrations (GMCs) and geometric mean titres (GMTs), respectively. We calculated 95% CIs for GMCs or GMTs by back transforming the 95% CI for log antibody concentrations or titres. We used a normal approximation method to calculate 95% CIs for proportions.

For the primary safety objective, the sample size was 25 participants per comparator group. For a serious event with a 0·01 rate of occurrence, the probability that zero participants will experience this event is 0·778 in each group. For the primary immunogenicity endpoint, a sample size of 25 per group would have 80% power to detect a 48% difference in response rates between two groups, if the true response rate in the unvaccinated group was 10%. Our sample size calculations were based on Fisher's exact test to compare the response rate between groups. Power calculations for this trial have been published.^15^ Additional post-hoc analyses examined immunogenicity in people with HIV who were SARS-CoV-2 seropositive before their first vaccine dose.

We used R (version 4.02) for all statistical analyses.^20^ The COV005 study is registered with ClinicalTrials.gov, NCT04444674, and the Pan African Clinical Trials Registry, PACTR202006922165132.

### Role of the funding source

The funders of the study had no role in study design, data collection, data analysis, data interpretation, or writing of the report. AstraZeneca reviewed the data from the trial and the final manuscript before submission and funded writing and editing assistance, but the academic authors retained editorial control.

## Results

**Figure 1:**
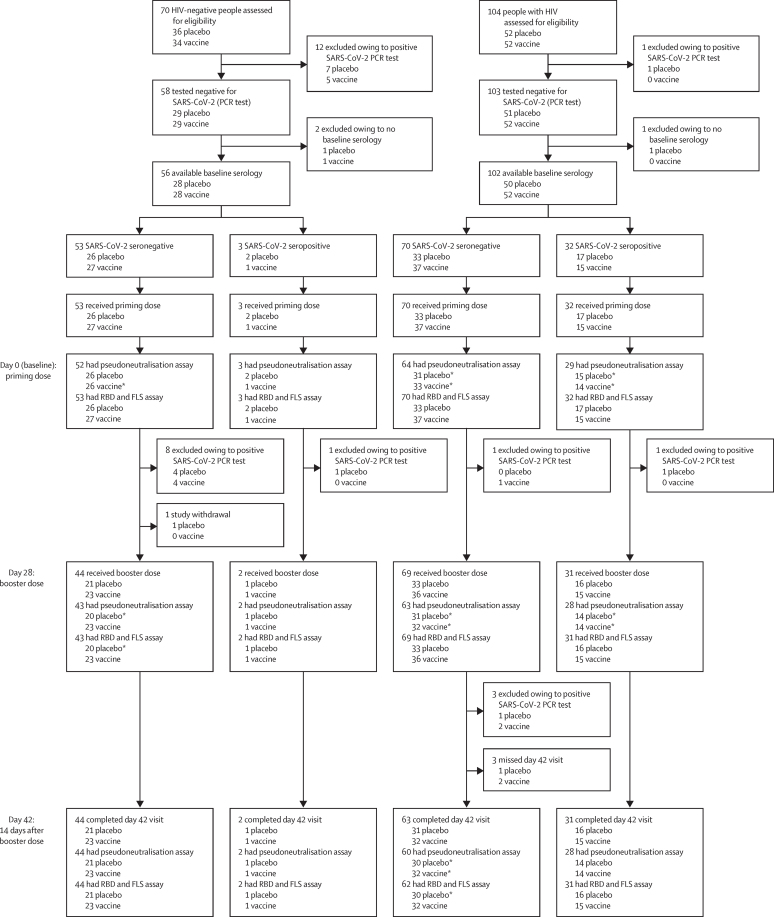
Study profile, stratified by SARS-CoV-2 serostatus 27 participants (21 HIV-negative people and six people with HIV) were excluded from this interim analysis because of a positive SARS-CoV-2 PCR test within the trial period. Three participants were excluded from the immunogenicity analysis owing to an absence of baseline serology. 12 participants (one withdrawn and 11 positive SARS-CoV-2 PCR tests) did not receive a booster dose. Three participants were excluded from analysis at day 42 as they were lost to follow-up. FLS=full length spike. RBD=receptor-binding domain. *One or more samples were not determined due to insufficient sample volume, haemolysis, or sample not collected.

We enrolled 104 people with HIV (between Aug 17 and Nov 12, 2020) and 70 HIV-negative individuals (between June 24 and July 29, 2020) to the intensive safety and immunogenicity cohort. One person with HIV and 12 HIV-negative participants had positive SARS-CoV-2 PCR tests at randomisation and were excluded from all analyses. One further person with HIV and two HIV-negative participants without baseline serology were excluded from immunogenicity analyses. 35 (22%) of 158 participants, including 32 (31%) of 102 people with HIV had serological evidence of previous SARS-CoV-2 infection (RBD IgG positive) before receipt of the priming dose of study treatment and were enrolled, as a result of high infection burden within the population. After excluding participants with positive SARS-CoV-2 PCR tests or unavailable serology results at baseline, 56 HIV-negative participants (28 vaccine; 28 placebo) and 102 people with HIV (52 vaccine; 50 placebo) received the priming dose of trial intervention (figure 1). 32 (31%) of 102 people with HIV and three (5%) of 56 HIV-negative participants tested seropositive (RBD IgG positive) for SARS-CoV-2 at randomisation; this difference is probably a result of different enrolment dates, as the former were enrolled after the first epidemic wave of COVID-19 and were, therefore, more likely to have been exposed to SARS-CoV-2.

Between the priming dose and before day 42 (14 days post-booster dose), an additional five people with HIV and nine HIV-negative participants were excluded from further analyses because of positive SARS-CoV-2 PCR tests (figure 1). One further HIV-negative participant in the placebo group was withdrawn from the trial (between doses) because of previously undisclosed history of mental illness, and three people with HIV (one placebo, two vaccine) missed day 42 trial visits.

In the overall population, excluding participants who had a positive SARS-CoV-2 PCR test but including participants with no baseline serology, 62% of HIV-negative participants were men, compared with 26% of people with HIV. 99% were Black (table). The median age of HIV-negative participants was lower than that of people with HIV. HIV-negative participants had a lower prevalence of underlying hypertension and chronic respiratory conditions, compared with people with HIV. The proportion of individuals with a body-mass index of 30·0–39·9 kg/m^2^ was similar between HIV-negative participants and people with HIV. People with HIV were stable on ART, with a median CD4 count of 695 cells per μL, a CD4 percentage of 36%, and 75% of participants had viral loads of less than 50 copies per mL (table).

**Table:** Baseline demographics

		**Overall (n=161)**	**HIV-negative participants**			**People with HIV**		
			Overall (n=58)	Placebo (n=29)	Vaccine (n=29)	Overall (n=103)	Placebo (n=51)	Vaccine (n=52)
Median (IQR) age, years		37 (31–44)	32 (25–42)	31 (26–42)	34 (23–41)	40 (33–46)	41 (36–46)	37 (32–45)
Sex								
	Female	98 (61%)	22 (38%)	10 (34%)	12 (41%)	76 (74%)	40 (78%)	36 (69%)
	Male	63 (39%)	36 (62%)	19 (66%)	17 (59%)	27 (26%)	11 (22%)	16 (31%)
Race								
	Black	160 (99%)	58 (100%)	29 (100%)	29 (100%)	102 (99%)	51 (100%)	51 (98%)
	White	1 (1%)	0	0	0	1 (1%)	0	1 (2%)
Body-mass index, kg/m^2^								
	<18	12 (8%)	7 (12%)	4 (14%)	3 (10%)	5 (5%)	1 (2%)	4 (8%)
	18–24·9	68 (42%)	26 (45%)	15 (52%)	11 (38%)	42 (41%)	18 (35%)	24 (46%)
	25–29·9	46 (29%)	13 (22%)	4 (14%)	9 (31%)	33 (32%)	16 (31%)	17 (33%)
	30–39·9	35 (22%)	12 (21%)	6 (21%)	6 (21%)	23 (22%)	16 (31%)	7 (14%)
Current smoker		61 (38%)	27 (47%)	12 (41%)	15 (52%)	34 (33%)	16 (31%)	18 (35%)
Current alcohol drinker		72 (45%)	25 (43%)	13 (45%)	12 (41%)	47 (46%)	21 (41%)	26 (50%)
Health-care worker		3 (2%)	1 (2%)	1 (3%)	0	2 (2%)	1 (2%)	1 (2%)
Hypertension		12 (8%)	1 (2%)	0	1 (3%)	11 (11%)	7 (14%)	4 (8%)
Chronic respiratory disease		16 (10%)	0	0	0	16 (16%)	10 (20%)	6 (12%)
HbA_1c_								
	Low	12 (8%)	2 (3%)	0	2 (7%)	10 (10%)	5 (10%)	5 (10%)
	Normal	145 (90%)	53 (91%)	26 (90%)	27 (93%)	92 (89%)	46 (90%)	46 (89%)
	High	4 (3%)	3 (5%)	3 (10%)	0	1 (1%)	0	1 (2%)
ART use*								
	NNRTI and two NRTIs	NA	NA	NA	NA	57 (76%)	29 (74%)	28 (78%)
	INSTI and two NRTIs	NA	NA	NA	NA	11 (15%)	5 (13%)	6 (17%)
	Boosted PI and one NRTI	NA	NA	NA	NA	4 (5%)	3 (8%)	1 (3%)
	Boosted PI and two NRTIs	NA	NA	NA	NA	3 (4%)	2 (5%)	1 (3%)
Years on ART								
	<1	NA	NA	NA	NA	9 (12%)	4 (10%)	5 (14%)
	1 to <5	NA	NA	NA	NA	28 (37%)	12 (31%)	16 (44%)
	≥5	NA	NA	NA	NA	38 (51%)	23 (59%)	15 (42%)
Median (IQR) CD4 count, cells per μL		NA	NA	NA	NA	695 (512–929)	677 (500–889)	742 (540–953)
Median (IQR) CD4 percentage		NA	NA	NA	NA	36 (30–41)	36 (29–41)	37 (32–41)
Viral load <50 copies per mL		27 (75%)	NA	NA	NA	27 (75%)	18 (82%)	9 (64%)
Median (IQR) time between doses, days		28 (27–28)	28 (28–28)	28 (28–28)	28 (28–28)	28 (25–28)	28 (26–28)	28 (23–28)
Median (IQR) time post-boost, days		14 (14–14)	14 (14–15)	14 (14–14)	14 (14–15)	14 (14–14)	14 (14–14)	14 (14–14)

Data are n (%) unless otherwise stated. Data exclude patients who were SARS-CoV-2 seropositive at baseline and include patients with no baseline serology available. ART=antiretroviral therapy. HbA_1c_=glycated haemoglobin. INSTI=integrase strand transfer inhibitor. NA=not applicable. NNRTI=non-nucleoside reverse transcriptase inhibitor. NRTI=nucleoside or nucleotide reverse transcriptase inhibitors. PI=protease inhibitors.

* Most participants (75%) were receiving an efavirenz-based regimen, 15% were receiving a dolutegravir-based regimen with two NRTIs (tenofovir and lamivudine or emtricitabine), and one participant received zidovidine and lamivudine. The remaining participants received a boosted protease inhibitor-based regimen, either lopinavir plus ritonavir or atazanavir plus ritonavir, with either one or two NRTIs, including lamivudine, zidovudine, abacavir, or tenofovir.

The demographic characteristics of participants who were SARS-CoV-2 seronegative at the time of randomisation and, therefore, eligible for inclusion in our post-booster-dose immune response analyses, and the demographic characteristics of participants who were SARS-CoV-2 seropositive at randomisation, were similar to those of the overall population (appendix pp 3–5). Demographic characteristics of people with HIV are in the appendix (p 6).

**Figure 2:**
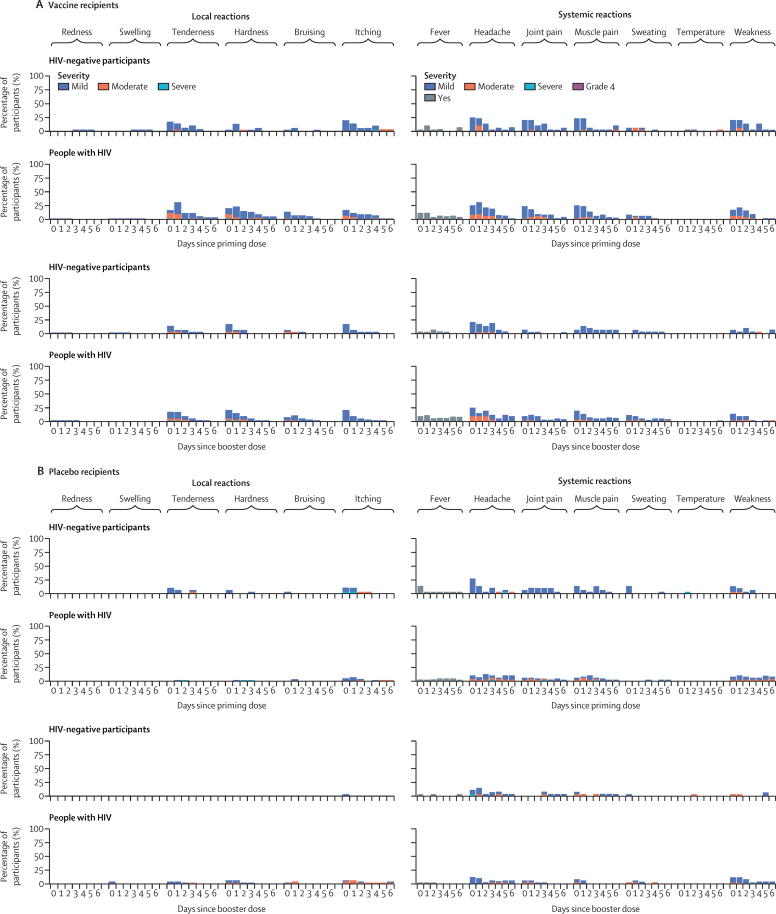
Solicited local and systemic adverse reactions in the 7 days after priming and booster doses of ChAdOx1 nCoV-19 Day 0 is the day of the priming dose. The severity of adverse events was graded as mild, moderate, or severe.

In HIV-negative participants and people with HIV, tenderness, hardness, bruising, and itching at injection site were the most commonly reported local reactions in vaccine and placebo recipients (figure 2; appendix pp 7–19). These events were less common among HIV-negative participants than in people with HIV and, in both groups, were predominantly mild or moderate in intensity, with moderate symptoms reported more often in the first 2 days after vaccination. Among both HIV-negative participants and people with HIV, there were no increases in reported local reactions after receiving the booster dose. Headache, joint and muscle pain, and weakness were the most commonly reported systemic reactions, occurring in almost a quarter of participants in the first 2 days after the priming dose (figure 2). Symptoms were mild or moderate in intensity over the first 48 h in all vaccinees. Fever, rigors, and sweating, lasting up to 7 days, were less commonly reported in HIV-negative participants than in people with HIV. There were 237 adverse events in HIV-negative participants (142 placebo; 95 vaccine) compared with 164 adverse events in people with HIV (78 placebo; 86 vaccine). The most common adverse event was general system disorders not elsewhere classified in both treatment groups in HIV-negative participants and people with HIV (appendix pp 20–23). Overall, seven serious adverse events occurred (appendix p 24). Six of these occurred in HIV-negative participants (four in those receiving the vaccine, two in those receiving placebo), of which five were deemed unrelated to the trial intervention. One HIV-negative participant reported a temperature of 40·5°C after the primary dose. However, the temperature resolved within 1 day with paracetamol, and no reactions to booster dose were reported. One person with HIV who received placebo treatment died during the study; this death was deemed unlikely to be related to the trial intervention.

There were few haematological abnormalities and no clinically important worsening was seen in haematology or chemistry panels in any of the trial groups (appendix pp 33–34). People with HIV had mild levels of alkaline phosphatase elevation in both vaccine and placebo groups throughout the trial. There were four potassium level increases graded as severe or higher because samples were haemolysed, and all subsequent blood draws showed normal levels of potassium.

Of the 32 participants with a positive SARS-CoV-2 PCR test during the trial, only one (a person with HIV) displayed moderate COVID-19 symptoms, which occurred between day 28 (28 days after priming dose) and day 42 (14 days after booster dose; appendix p 25). The remaining participants with a positive SARS-CoV-2 PCR test were asymptomatic (six HIV-negative participants and one person with HIV), had mild symptoms (eight HIV-negative participants and five people with HIV), or had other symptoms that did not meet the protocol definition of mild COVID-19 disease (ten HIV-negative participants and one person with HIV).

**Figure 3:**
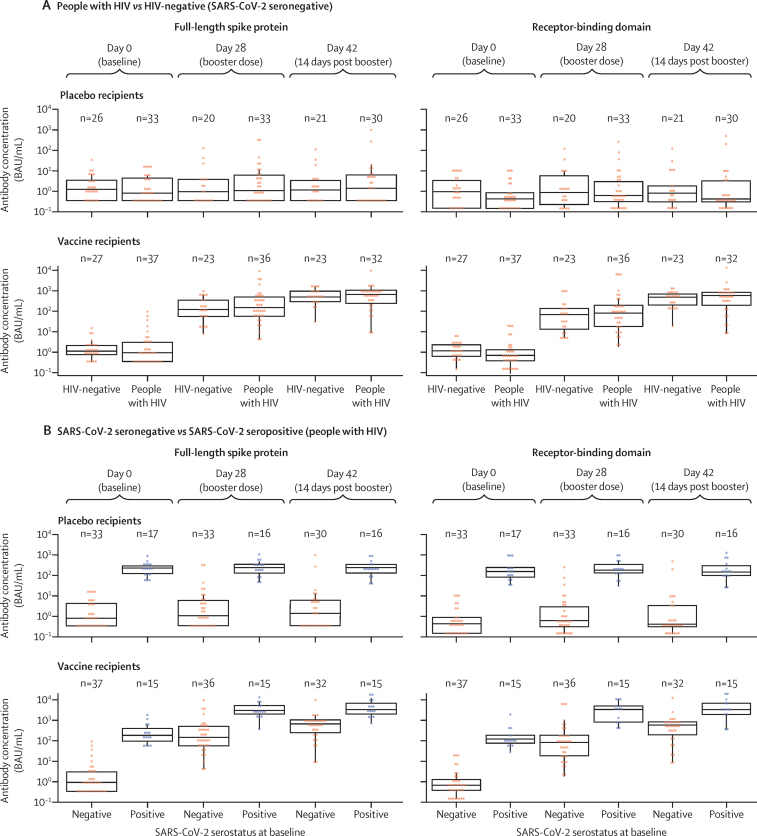
Immunogenicity to SARS-CoV-2 full-length spike and receptor-binding domain proteins Analyses stratified by HIV status (A) and by SARS-CoV-2 serostatus (B) at baseline in people with HIV. Antibody responses assessed at day 0 (baseline), day 28 (post-priming dose), and day 42 (14 days post-booster dose). Boxes denote interquartile ranges and horizontal bars denote median antibody concentration in BAU/mL. BAU=binding antibody unit.

Primary immunogenicity analyses in SARS-CoV-2 seronegative participants included samples from 44 HIV-negative participants (21 placebo, 23 vaccine) and 62 people with HIV (30 placebo, 32 vaccine). Immunised participants showed a strong, vaccine-induced serum IgG response against FLS and RBD, regardless of HIV status, which increased with the booster dose (figure 3A). 28 days after the priming dose, the median FLS IgG GMC was 163·7 BAU/mL (95% CI 89·9–298·1) for people with HIV (n=36) and 112·3 BAU/mL (61·7–204·4) for HIV-negative participants (n=23). A booster response was measured at day 42, with a median GMC of 453·1 BAU/mL (267·4–767·7) in people with HIV (n=32), and 504·9 BAU/mL (337·1–756·2) in HIV-negative participants (n=23; appendix p 26). Similar IgG response patterns were seen for ChAdOx1 nCoV-19-induced geometric mean RBD-binding IgG concentrations (appendix p 27). Seropositivity for either FLS or RBD IgG was similar in people with HIV and HIV-negative participants who received the vaccine. At day 28, seropositivity for FLS IgG was 86% (95% CI 71·3–93·9) in people with HIV and 78% (58·1–90·3) in HIV-negative participants (appendix p 26).

**Figure 4:**
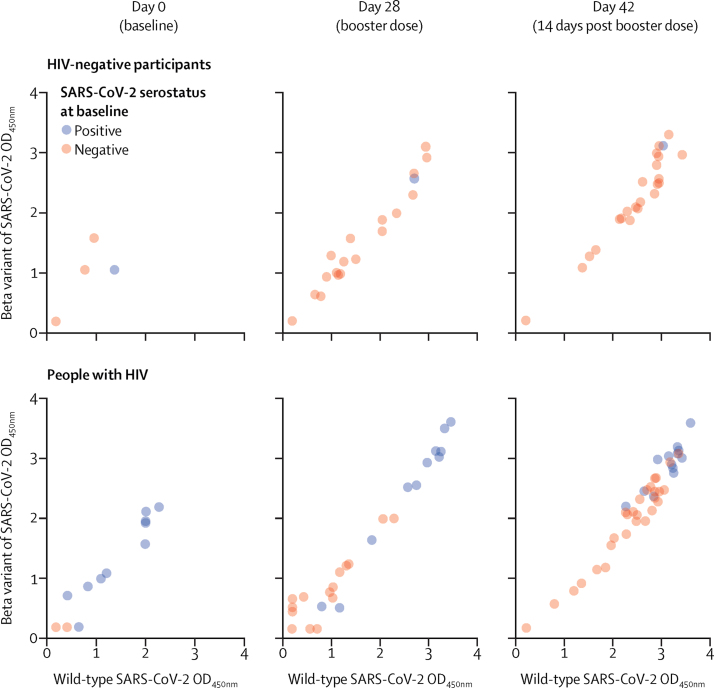
Correlation of plasma IgG antibody binding to Wuhan-1 Asp614Gly wild-type *vs* beta (B.1.351) full-length spike protein in vaccine recipients Analyses stratified by day and HIV status. OD=optical density.

We also assessed immunogenicity in people with HIV based on SARS-CoV-2 serostatus at baseline, excluding patients with a positive SARS-CoV-2 PCR test, enabling a post-hoc analysis of immune responses in this group after vaccination (figure 3B). The same analysis was not possible for the HIV-negative group because only three participants were SARS-CoV-2 seropositive; although six HIV-negative participants tested seropositive for SARS-CoV-2, three were excluded because they had a positive SARS-CoV-2 PCR test at the time of randomisation. At day 28, both FLS and RBD IgG concentrations increased substantially from baseline after the first dose, with modest increases seen by day 42 (appendix pp 28–29). The IgG GMCs after the priming dose of ChAdOx1-nCoV-19 in people with HIV who were SARS-CoV-2 seropositive at enrolment were 18·0–29·7-times higher after the priming dose and 6·5–6·8-times higher after the booster dose in people with HIV who were SARS-CoV-2 naive at baseline (appendix pp 28–29). When assessing cross-reactivity of binding antibodies to the FLS from the beta variant, vaccinated people with HIV and HIV-negative participants showed strong correlations in their ability to bind FLS from Asp614Gly wild-type and the beta variant, regardless of baseline SARS-CoV-2 serostatus (figure 4). Corresponding plasma IgG antibody binding data for placebo recipients is given in the appendix (appendix p 35).

In the 26 HIV-negative participants vaccinated with ChAdOx1 nCoV-19 who were assessed for neutralisation activity against Asp614Gly wild-type, the GMT of SARS-CoV-2 neutralising antibodies strongly correlated with Asp614Gly wild-type antigen-specific IgG GMCs on days 28 and 42. Of the 25 HIV-negative, SARS-CoV-2 baseline-seronegative participants, two mounted neutralising responses at day 0, with GMT neutralisation activity of inhibitory dilution 50% (ID_50_) 31·2 (95% CI 1·9–526·6), and 13 (59%) of 22 participants mounted neutralising responses of ID_50_ 135·0 (54·5–334·2) by day 28. Neither of the two participants who showed neutralisation activity at day 0 subsequently showed an increase of at least two times at day 28. At day 42, 20 participants mounted neutralising responses with GMT neutralising activity ID_50_ 316·4 (184·8–541·8; appendix p 30).

**Figure 5:**
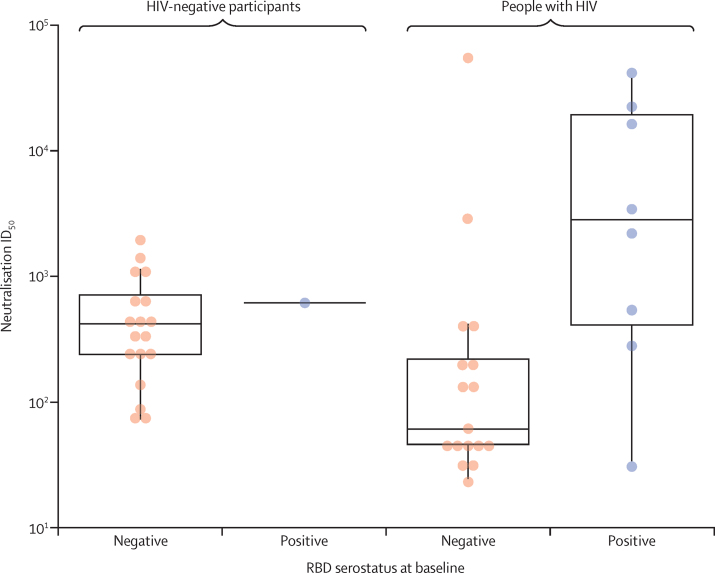
Pseudovirus neutralisation responses to Wuhan-1 Asp614Gly wild-type on day 42 in vaccinees Samples below the limit of detection are not shown. Boxes denote interquartile ranges, and horizontal bars denote neutralisation ID_50_. ID_50_=inhibitory dilution (50%). RBD=receptor-binding domain.

Using an MLV-based neutralisation assay, we also assessed neutralising antibody activity against Asp614Gly wild-type in all samples from people with HIV who were RBD seropositive at day 42. Of these, data were obtained for 33 (65%) samples. Of the 18 people with HIV who were RBD seronegative at baseline and received the vaccine, 17 mounted neutralising responses, with point estimate GMT neutralisation activity of ID_50_ 151·5 (95% CI 954·8–419·0) being lower than that of 18 HIV-negative participants who were RBD seronegative at baseline with neutralisation activity of ID_50_ of 394·2 (242·0–642·1; figure 5); albeit with overlapping 95% CI.

Among 13 HIV-negative vaccinees with neutralisation activity against Asp614Gly wild-type, only two participants retained activity against the beta variant. By contrast, among 20 people with HIV with neutralisation activity against Asp614Gly wild-type, ten vaccinees retained activity against the beta variant, eight of whom were seropositive for SARS-CoV-2 at baseline (appendix p 36). Corresponding neutralisation activity in people with HIV who received the placebo is provided in the appendix (p 37).

## Discussion

Results of this interim phase 1B/2A analysis, show that two doses of ChAdOx1 nCoV-19 were well tolerated, with similar FLS-binding and RBD-binding IgG and SARS-CoV-2 neutralising response patterns in people with HIV and HIV-negative SARS-CoV-2-naive participants after priming and booster doses of the vaccine. Our findings are similar to previously reported immunogenicity of this vaccine across all adult age groups.^4^

Antibody responses to FLS and RBD viral proteins induced by each dose were also seen in people with HIV who had previously been exposed to SARS-CoV-2. Notably, previous infection with SARS-CoV-2 in people with HIV was associated with robust immune responses after the first dose of ChAdOx1 nCoV-19, with GMCs exceeding by 6·49–6·84 times those seen post-booster dose in vaccine recipients who were seronegative at enrolment, as similarly reported for mRNA vaccines, BNT162b2 (Pfizer–BioNTech) and mRNA-1273 (Moderna) in HIV-negative participants.^21^

In our trial, development of neutralising titres against the Asp614Gly wild-type strain after priming and booster doses of ChAdOx1 nCoV-19 correlated with antibody responses to FLS and RBD viral antigens in HIV-negative participants and in people with HIV. Taken together, these findings suggest that vaccination with ChAdOx1 nCoV-19 might consolidate the immune response and drive long-term immune memory, providing a protective benefit to this population against the ancestral strain, regardless of previous SARS-CoV-2 exposure. Neutralising antibodies have been implicated as correlates of protection from COVID-19 in preclinical challenge studies^22^ and, in a previous clinical trial, neutralising antibodies developed against the Asp614Gly wild-type strain in more than 99% of participants after vaccination with ChAdOx1 nCoV-19, with higher levels in boosted than in non-boosted groups.^4^ However, to date, no correlates of protection have been defined from clinical COVID-19 vaccine studies.

The majority of adverse events reported in people with HIV and HIV-negative participants were mild or moderate in intensity, which is consistent with the reported safety profile of ChAdOx1 nCoV-19.^1^, ^6^, ^14^ Reactogenicity, which we assessed 7 days after the priming and booster doses of vaccine, was lower in HIV-negative participants than in people with HIV, with symptoms that were predominantly mild or moderate in intensity. In both populations, fewer adverse events were reported after the booster dose than after the priming dose and no clinically important worsening was seen in haematology or chemistry panels compared with placebo.

People with HIV have a greater risk for fatal outcome upon COVID-19-related admission to hospital^8^ with higher associations in people of Black ethnicity than in non-Black individuals.^23^ In South Africa, HIV infection has also been associated with an increased risk of death from COVID-19, irrespective of HIV-1 viral load and immunosuppression.^24^

Conducting vaccine trials in Africa is vital to ensure that COVID-19 vaccines will be effective in this setting, particularly given the burden of HIV in sub-Saharan Africa^13^ and the emergence of variants such as the beta variant.^5^ In the overall analysis of the present phase 1B/2A trial, published elsewhere,^6^ the two-dose regimen of ChAdOx1 nCoV-19 did not show protection against non-hospitalised mild to moderate COVID-19 after infection with the beta variant in young HIV-negative adults in South Africa. In those participants, ChAdOx1 nCoV-19 induced strong neutralising antibodies 28 days after the first dose, which increased after booster dose was given 21–35 days later, yet neutralising activity was reduced or undetected against the beta variant.^6^ Although efficacy against severe disease caused by beta could not be assessed, in a preclinical study in hamsters, vaccination with ChAdOx1 nCoV-19 protected against pneumonia when animals were challenged with beta, which is consistent with protection against severe disease but not mild disease restricted to the upper respiratory tract.^25^

In this interim analysis, we show that, in people with HIV and HIV-negative vaccinees, vaccine-elicited binding antibodies to FLS show high level cross-reactivity for the beta variant. These results are similar to previously published results from convalescent donor plasma.^17^, ^26^ Binding antibodies might contribute to Fc effector functions, which have been implicated in preventing severe disease in convalescent donors. Whether this effect is also true of cross-reactive vaccine-elicited binding antibodies remains to be determined.

Furthermore, in those vaccinated people with HIV who developed neutralising responses to Asp614Gly wild-type, neutralisation against the beta variant was retained in 50% of vaccinated participants, 80% of whom were RBD IgG seropositive at baseline, and in whom ChAdOx1 nCoV-19 induced high neutralising antibody titres against the original Asp614Gly wild-type strain. These observations suggest that ChAdOx1 nCoV-19 vaccination in settings with a high prevalence of SARS-CoV-2 infection due to the ancestral virus could result in some protection, even against mild to moderate COVID-19 caused by the beta variant.

Our study has some limitations. First, the small sample size of HIV-negative participants who were SARS-CoV-2 seropositive, which precluded comparisons between SARS-CoV-2 seropositive participants in both groups. Second, experiments on cell-mediated immune responses in participants have not yet been completed. Previous studies have shown that spike-specific T-cell responses are induced by ChAdOx1 nCoV-19 in vaccine recipients from 7 days after the priming dose.^4^ Third, owing to the phase 1B/2A design of this trial, a relatively risk-averse group of people with HIV receiving ART and who were virally suppressed with CD4 counts of more than 500 cells per μL were included, limiting its generalisability to the overall population of people with HIV. Finally, ART can interfere with the performance of neutralisation assays, which explains the relatively low number of people with HIV whose sera provided neutralising antibody data in this trial.

Given the prevalence of emerging SARS-CoV-2 variants of concern, further trials in people with HIV and in South Africa, which include analyses of cellular responses, are warranted, as are additional studies to correlate vaccine-induced immunogenicity with COVID-19 protection. However, the findings from this interim analysis of administration of a COVID-19 vaccine specific to people with HIV, and specific to Africa, show favourable safety and immunogenicity of ChAdOx1 nCoV-19. Our findings also suggest that previous exposure to the ancestral SARS-CoV-2 virus, Asp614Gly wild-type, in people with HIV might result in a heightened immune response, including some preservation of neutralising antibody activity against the beta variant.

## Data sharing

Data underlying the findings described in this manuscript can be obtained in accordance with AstraZeneca's data sharing policy described at https://astrazenecagrouptrials.pharmacm.com//ST/Submission/Disclosure. Anonymised participant data will be made available when the trial is complete, upon request directed to the corresponding author. Proposals will be reviewed and approved by the sponsor, investigator, and collaborators on the basis of scientific merit. Upon approval of a proposal, data can be shared through a secure online platform after signing a data access agreement. All data will be made available for a minimum of 5 years from the end of the trial. Full details of the approved trial protocol (version 6.0) are available online.^15^

## Declaration of interests

Oxford University has entered into a partnership with AstraZeneca for further development of ChAdOx1 nCoV-19 (AZD1222). AstraZeneca reviewed the data from the trial and the final manuscript before submission, but the authors retained editorial control. SCG is cofounder of Vaccitech, a collaborator in the early development of this vaccine candidate, and is named as an inventor on a patent covering use of ChAdOx1-vectored vaccines (PCT/GB2012/000467) and a patent application covering this SARS-CoV-2 vaccine (GB2003670.3). TL is named as an inventor on a patent application covering ChAdOx1 nCoV-19 and was a consultant to Vaccitech. TLV and JV are employees of AstraZeneca. All other authors declare no competing interests.
