# Correction to *Lancet HIV* 2021; 8: e568–80

**DOI:** 10.1016/S2352-3018(22)00140-0

**Published:** 2022-05-18

*Madhi SA, Koen AL, Izu A, et al. Safety and immunogenicity of the ChAdOx1 nCoV-19 (AZD1222) vaccine against SARS-CoV-2 in people living with and without HIV in South Africa: an interim analysis of a randomised, double-blind, placebo-controlled, phase 1B/2A trial.* Lancet HIV *2021; **8:** e568–80*—In the Summary of this Article, the fifth sentence of the Findings section should have read “One HIV-negative participant died (unlikely related)”. The final sentence of the fifth paragraph in the Results section should have read “One HIV-negative participant who received placebo treatment died during the study;…”. These corrections have been made to the online version as of May 18, 2022.

