# Supplementary material for: Safety and immunogenicity of the ChAdOx1 nCoV-19 (AZD1222) vaccine against SARS-CoV-2 in people living with and without HIV in South Africa: an interim analysis of a randomised, double-blind, placebo-controlled, phase 1B/2A trial
Source: Lancet HIV. 2021 Aug 17;8(9):e568–80. doi: 10.1016/S2352-3018(21)00157-0 (PMC8372504; doi:10.1016/S2352-3018(21)00157-0)
Supplement: Supplementary appendix [file mmc1.pdf]

THE LANCET
HIV

Supplementary appendix

This appendix formed part of the original submission and has been peer reviewed. We post it as supplied by the authors.

Supplement to: Madhi S A, Koen A L, Izu A, et al. Safety and immunogenicity of the ChAdOx1 nCoV-19 (AZD1222) vaccine against SARS-CoV-2 in people living with and without HIV in South Africa: an interim analysis of a randomised, double-blind, placebo-controlled, phase 1B/2A trial. *Lancet HIV* 2021; published online August 17. http://dx.doi.org/10.1016/S2352-3018(21)00157-0.

Supplementary Material

Methods

Luminex assay sensitivity and specificity

Serum samples collected prior to 2020 (n=31) were used to determine assay specificity. Cut-offs of 32 binding antibody units (BAU)/mL and 26 BAU/mL for full length spike (FLS) and receptor-binding domain (RBD) immunoglobulin G (IgG), respectively, were selected as thresholds indicating severe acute respiratory syndrome coronavirus 2 (SARS-CoV-2) infection, based on the highest values measured in pre-coronavirus disease 2019 (COVID-19) samples. Clinical assay sensitivity for detecting past or current infection was assessed using serum samples obtained from randomly selected (n=15) participants who tested positive for SARS-CoV-2 by PCR and who had serial sampling before and after symptom onset, including cases with mild to moderate illness and asymptomatic infections. Clinical sensitivity of the IgG assay for both FLS and RBD was 75% for samples taken at 7–14 days following a positive SARS-CoV-2 PCR result, and 100% for samples taken after 14 days. The optimal serum/plasma and secondary antibody dilutions for the assay were 1:100 and 1:200, respectively. Samples exceeding the dynamic range of the assay were re-tested at higher dilutions (1:200–1:1000). Samples were analysed in true duplicate and each plate included two in-house control sera. Bead fluorescence was read with the Bio-Plex 200 instrument (Bio-Rad) using Bio-Plex manager 5·0 software (Bio-Rad).

ELISA

Recombinant proteins were coated onto 96-well, high-binding plates, and incubated overnight at 4°C. Plates were washed and blocked at 37°C for 1–2 hours. Plasma samples from people living with HIV (PLWH) and HIV-negative participants from days 0, 28 and 42 were diluted and tested by incubating at 37°C in the 96-well plates for 1 hour, followed by incubation with anti-human horseradish peroxidase-conjugated antibody and signal development with tetramethylbenzidine chromogenic substrate (ThermoFisher Scientific). Absorbance at 450 nm was measured for each 96-well plate. In all instances, monoclonal antibodies, CR3022 or palivizumab, were used as positive and negative controls, respectively. Data are plotted as area under the curve.

Vaccine manufacturing

Three batches of ChAdOx1 nCoV-19 (AZD1222) were used in this study. Two batches (batch K·0008 and batch K·0011) were manufactured and vialed by Advent (Pomezia, Italy), and the third (batch A03367) was produced by COBRA Biologics (Keele, United Kingdom) and vialed by Symbiosis (Sterling, United Kingdom). According to analytical analysis, vaccines from all lots were not expected to have clinically significant differences in reactogenicity, immunogenicity, or efficacy. All vaccine batches were manufactured according to Good Manufacturing Practice and approved by the Medicines and Healthcare Products regulatory agency in the United Kingdom. Of the participants included in the safety analysis, 34, 32, and 15 received batches K·0008, K·0011, and

A03367 for their priming dose, respectively. For the booster dose, 28 received batch K·0008, 25 received batch K·0011, and 28 received batch A03367.

Supplementary Table 1. Baseline demographics for participants who were SARS-CoV-2 seronegative at baseline, stratified by HIV status.

Characteristic	Overall	HIV-negative participants			PLWH		
	(N=123)	Overall (n=53)	Placebo (n=26)	Vaccine (n=27)	Overall (n=70)	Placebo (n=33)	Vaccine (n=37)
Median (IQR) age, years	36 (31–44)	33 (25–43)	32 (26–44)	34 (23–42)	38 (33–46)	40 (36–46)	35 (32–45)
Male, n (%)	52 (42·3)	32 (60·4)	16 (61·5)	16 (59·3)	20 (28·6)	8 (24·2)	12 (32·4)
Race, n (%)							
Black	122 (99·2)	53 (100)	26 (100)	27 (100)	69 (98·6)	33 (100)	36 (97·3)
White	1 (0·8)	0	0	0	1 (1·4)	0	1 (2·7)
BMI, kg/m^2, n (%)							
<18	10 (8·1)	6 (11·3)	4 (15·4)	2 (7·4)	4 (5·7)	1 (3)	3 (8·1)
18–24·9	52 (42·3)	24 (45·3)	13 (50)	11 (40·7)	28 (40)	12 (36·4)	16 (43·2)
25–29·9	36 (29·3)	11 (20·8)	3 (11·5)	8 (29·6)	25 (35·7)	11 (33·3)	14 (37·8)
30–39·9	25 (20·3)	12 (22·6)	6 (23·1)	6 (22·2)	13 (18·6)	9 (27·3)	4 (10·8)
Smoker, n (%)	50 (40·7)	25 (47·2)	12 (46·2)	13 (48·1)	25 (35·7)	11 (33·3)	14 (37·8)
Alcohol, n (%)	50 (40·7)	23 (43·4)	13 (50)	10 (37)	27 (38·6)	10 (30·3)	17 (45·9)
Health care worker, n (%)	3 (2·4)	1 (1·9)	1 (3·8)	0	2 (2·9)	1 (3)	1 (2·7)
Hypertension, n (%)	7 (5·7)	1 (1·9)	0	1 (3·7)	6 (8·6)	2 (6·1)	4 (10·8)
Respiratory system, n (%)	10 (8·1)	0 (0)	0	0	10 (14·3)	7 (21·2)	3 (8·1)
HbA1c, n (%)							
Low	8 (6·5)	2 (3·8)	0	2 (7·4)	6 (8·6)	3 (9·1)	3 (8·1)
Normal	111 (90·2)	48 (90·6)	23 (88·5)	25 (92·6)	63 (90)	30 (90·9)	33 (89·2)
High	4 (3·3)	3 (5·7)	3 (11·5)	0	1 (1·4)	0	1 (2·7)
ARV, n (%)*							
NNRTI and 2 NRTIs	NA	NA	NA	NA	41 (73·2%)	19 (70·4%)	22 (75·9%)
INSTI and 2 NRTIs	NA	NA	NA	NA	9 (16·1%)	4 (14·8%)	5 (17·2%)
Boosted PI and 1 NRTI	NA	NA	NA	NA	3 (5·4%)	2 (7·4%)	1 (3·4%)
Boosted PI and 2 NRTIs	NA	NA	NA	NA	3 (5·4%)	2 (7·4%)	1 (3·4%)
Time on ARV, n (%)							
<1 year	NA	NA	NA	NA	6 (10·7%)	3 (11·1%)	3 (10·3%)
1–<5 years	NA	NA	NA	NA	21 (37·5%)	9 (33·3%)	12 (41·4%)
≥5 years	NA	NA	NA	NA	29 (51·8%)	15 (55·6%)	14 (48·3%)
Median (IQR) CD4+ count, cells/µL	NA	NA	NA	NA	690 (503–940)	593 (498–900)	746 (554–952)
Median (IQR) CD4+ percentage	NA	NA	NA	NA	35 (30–41)	34 (29–39)	36 (32–41)
Median (IQR) detectable viral load, copies/mL	NA	NA	NA	NA	10 (10–54)	10 (10–26)	30 (10–96)
Median (IQR) time between doses, days	28 (27–28)	28 (28–28)	28 (28–28)	28 (28–28)	28 (25–28)	28 (27–28)	28 (23–28)
Median (IQR) time post-boost, days	14 (14–14)	14 (14–15)	14 (14–14)	14 (14–15)	14 (14–14)	14 (14–14)	14 (14–14)

*Most (75%) were receiving an efavirenz-based regimen and 14·5% were receiving a dolutegravir-based regimen with two nucleoside and nucleotide reverse transcriptase inhibitors (NRTIs), tenofovir and lamivudine or emtricitabine, and one participant received zidovidine and lamivudine. The remaining participants received a boosted protease inhibitor-based regimen, either lopinavir/ritonavir or atazanavir/ritonavir, with either one or two NRTIs, including lamivudine, zidovudine, abacavir, or tenofovir.

ARV= antiretroviral treatment; BMI=body mass index; HbA1c=glycated haemoglobin measurement (indicator of diabetes); HIV=human immunodeficiency virus; INSTI=integrase strand transfer inhibitor; IQR=interquartile range; n=subpopulation number; NA=not applicable; NNRTI=non-nucleoside reverse transcriptase inhibitor; NRTI= nucleoside and nucleotide reverse transcriptase inhibitors; PI= protease inhibitor; PLWH=people living with HIV; SARS-CoV-2=severe acute respiratory syndrome coronavirus 2.

Supplementary Table 2. Baseline demographics for participants who were SARS-CoV-2 seropositive at baseline, stratified by HIV status.

Characteristic	Overall	HIV-negative participants			PLWH		
	(N=35)	Overall (n=3)	Placebo (n=2)	Vaccine (n=1)	Overall (n=32)	Placebo (n=17)	Vaccine (n=15)
Median (IQR) age, years	42 (33–47)	31 (31–34)	31 (31–31)	37 (37–37)	43 (36–47)	43 (40–47)	42 (31–47)
Male, n (%)	8 (22·9)	2 (66·7)	2 (100)	0	6 (18·8)	2 (11·8)	4 (26·7)
Race, n (%)							
Black	35 (100)	3 (100)	2 (100)	1 (100)	32 (100)	17 (100)	15 (100)
BMI, kg/m^2, n (%)							
<18	1 (2·9)	0	0	0	1 (3·1)	0	1 (6·7)
18–24.9	14 (40)	1 (33·3)	1 (50)	0	13 (40·6)	5 (29·4)	8 (53·3)
25–29.9	10 (28·6)	2 (66·7)	1 (50)	1 (100)	8 (25)	5 (29·4)	3 (20)
30–39.9	10 (28·6)	0	0	0	10 (31·2)	7 (41·2)	3 (20)
Smoker, n (%)	9 (25·7)	1 (33·3)	0	1 (100)	8 (25)	4 (23·5)	4 (26·7)
Alcohol, n (%)	20 (57·1)	1 (33·3)	0	1 (100)	19 (59·4)	10 (58·8)	9 (60)
Health care worker, n (%)	0	0	0	0	0	0	0
Hypertension, n (%)	5 (14·3)	0	0	0	5 (15·6)	5 (29·4)	0
Respiratory system, n (%)	6 (17·1)	0	0	0	6 (18·8)	3 (17·6)	3 (20)
HbA1c, n (%)							
Low	4 (11·4)	0	0	0	4 (12·5)	2 (11·8)	2 (13·3)
Normal	31 (88·6)	3 (100)	2 (100)	1 (100)	28 (87·5)	15 (88·2)	13 (86·7)
High	0	0	0	0	0	0	0
ARV, n (%)*							
NNRTI and 2 NRTIs	NA	NA	NA	NA	15 (83·3%)	9 (81·8%)	6 (85·7%)
INSTI and 2 NRTIs	NA	NA	NA	NA	2 (11·1%)	1 (9·1%)	1 (14·3%)
Boosted PI and 1 NRTI	NA	NA	NA	NA	1 (5·6%)	1 (9·1%)	0 (0%)
Boosted PI and 2 NRTIs	NA	NA	NA	NA	0 (0%)	0 (0%)	0 (0%)
Time on ARV, n (%)							
<1 year	NA	NA	NA	NA	3 (16·7%)	1 (9·1%)	2 (28·6%)
1–<5 years	NA	NA	NA	NA	7 (38·9%)	3 (27·3%)	4 (57·1%)
≥5 years	NA	NA	NA	NA	8 (44·4%)	7 (63·6%)	1 (14·3%)
Median (IQR) CD4+ count, cells/µL	NA	NA	NA	NA	714 (522–914)	731 (532–869)	696 (521–946)
Median (IQR) CD4+ percentage	NA	NA	NA	NA	37 (31–41)	37 (28–40)	37 (33–40)
Median (IQR) detectable viral load, copies/mL	NA	NA	NA	NA	16 (10–43)	10 (10–28)	46 (28–94)
Median (IQR) time between doses, days	28 (26–28)	28 (28–28)	28 (28–28)	27 (27–27)	28 (25–28)	28 (25–28)	28 (24–28)
Median (IQR) time post-boost, days	14 (14–14)	15 (14–16)	16 (15–16)	14 (14–14)	14 (14–14)	14 (14–14)	14 (14–14)

*Most (75%) were receiving an efavirenz-based regimen and 14·5% were receiving a dolutegravir-based regimen with two nucleoside and nucleotide reverse transcriptase inhibitors (NRTIs), tenofovir and lamivudine or emtricitabine, and one participant received zidovidine and lamivudine. The remaining participants received a boosted protease inhibitor-based regimen, either lopinavir/ritonavir or atazanavir/ritonavir, with either one or two NRTIs, including lamivudine, zidovudine, abacavir, or tenofovir.

ARV= antiretroviral treatment; BMI=body mass index; HbA1c=glycated haemoglobin measurement (indicator of diabetes); HIV=human immunodeficiency virus; INSTI=integrase strand transfer inhibitor; IQR=interquartile range; n=subpopulation number; NA=not applicable; NNRTI=non-nucleoside reverse transcriptase inhibitor; NRTI= nucleoside and nucleotide reverse transcriptase inhibitors; PI=protease inhibitor; PLWH=people living with HIV; SARS-CoV-2=severe acute respiratory syndrome coronavirus 2.

Supplementary Table 3. Baseline demographics for PLWH, stratified by SARS-CoV-2 serostatus at baseline and treatment group

Characteristic	SARS-CoV-2 seronegative			SARS-CoV-2 seropositive		
	**Overall (n=70)**	**Placebo (n=33)**	**Vaccine (n=37)**	**Overall (n=32)**	**Placebo (n=17)**	**Vaccine (n=15)**
Median (IQR) age, years	38 (33–46)	40 (36–46)	35 (32–45)	43 (36–47)	43 (40–47)	42 (31–47)
Male, n (%)	20 (28·6)	8 (24·2)	12 (32·4)	6 (18·8)	2 (11·8)	4 (26·7)
Race, n (%)						
Black	69 (98·6)	33 (100)	36 (97·3)	32 (100)	17 (100)	15 (100)
White	1 (1·4)	0 (0)	1 (2·7)	0 (0)	0 (0)	0 (0)
BMI, kg/m^2, n (%)						
<18	4 (5·7)	1 (3)	3 (8·1)	1 (3·1)	0 (0)	1 (6·7)
18–24·9	28 (40)	12 (36·4)	16 (43·2)	13 (40·6)	5 (29·4)	8 (53·3)
25–29·9	25 (35·7)	11 (33·3)	14 (37·8)	8 (25)	5 (29·4)	3 (20)
30–39·9	13 (18·6)	9 (27·3)	4 (10·8)	10 (31·2)	7 (41·2)	3 (20)
Smoker, n (%)	25 (35·7)	11 (33·3)	14 (37·8)	8 (25)	4 (23·5)	4 (26·7)

*Most (75%) were receiving an efavirenz-based regimen and 14·5% were receiving a dolutegravir-based regimen with two nucleoside and nucleotide reverse transcriptase inhibitors (NRTIs), tenofovir and lamivudine or emtricitabine, and one participant received zidovidine and lamivudine. The remaining participants received a boosted protease inhibitor-based regimen, either lopinavir/ritonavir or atazanavir/ritonavir, with either one or two NRTIs, including lamivudine, zidovudine, abacavir, or tenofovir.

ARV= antiretroviral treatment; BMI=body mass index; HbA1c=glycated haemoglobin measurement (indicator of diabetes); HIV=human immunodeficiency virus; INSTI=integrase strand transfer inhibitor; IQR=interquartile range; n=subpopulation number; NA=not applicable; NNRTI=non-nucleoside reverse transcriptase inhibitor; NRTI= nucleoside and nucleotide reverse transcriptase inhibitors; PI=protease inhibitor; PLWH=people living with HIV; SARS-CoV-2=severe acute respiratory syndrome coronavirus 2.

Supplementary Table 4. Solicited local and systemic AEs in the 7 days after priming and booster doses stratified by HIV status and treatment group

AE, n/N (%)	Day	Severity	HIV-negative participants		PLWH	
			Placebo	Vaccine	Placebo	Vaccine
**After priming dose**						
Ageusia	0	Mild	0/50 (0)	0/52 (0)	3/28 (10.7)	1/29 (3.4)
		Moderate	0/50 (0)	0/52 (0)	0/28 (0)	0/29 (0)
		Severe	0/50 (0)	0/52 (0)	0/28 (0)	0/29 (0)
	1	Mild	0/50 (0)	1/52 (1.9)	1/29 (3.4)	1/29 (3.4)
		Moderate	0/50 (0)	0/52 (0)	0/29 (0)	0/29 (0)
		Severe	0/50 (0)	0/52 (0)	0/29 (0)	0/29 (0)
	2	Mild	0/50 (0)	0/52 (0)	0/29 (0)	1/29 (3.4)
		Moderate	0/50 (0)	0/52 (0)	0/29 (0)	0/29 (0)
		Severe	0/50 (0)	0/52 (0)	0/29 (0)	0/29 (0)
	3	Mild	0/50 (0)	0/52 (0)	0/29 (0)	0/29 (0)
		Moderate	0/50 (0)	0/52 (0)	0/29 (0)	0/29 (0)
		Severe	0/50 (0)	0/52 (0)	0/29 (0)	0/29 (0)
	4	Mild	0/50 (0)	0/52 (0)	0/29 (0)	0/29 (0)
		Moderate	0/50 (0)	0/52 (0)	0/29 (0)	0/29 (0)
		Severe	0/50 (0)	0/52 (0)	0/29 (0)	0/29 (0)
	5	Mild	0/50 (0)	0/51 (0)	0/29 (0)	0/29 (0)
		Moderate	0/50 (0)	0/51 (0)	0/29 (0)	0/29 (0)
		Severe	0/50 (0)	0/51 (0)	0/29 (0)	0/29 (0)
	6	Mild	0/49 (0)	0/51 (0)	0/29 (0)	0/29 (0)
		Moderate	0/49 (0)	0/51 (0)	0/29 (0)	0/29 (0)
		Severe	0/49 (0)	0/51 (0)	0/29 (0)	0/29 (0)
Anosmia	0	Mild	0/50 (0)	0/52 (0)	0/29 (0)	2/29 (6.9)
		Moderate	0/50 (0)	0/52 (0)	0/29 (0)	0/29 (0)
		Severe	0/50 (0)	0/52 (0)	0/29 (0)	0/29 (0)
	1	Mild	0/50 (0)	0/52 (0)	0/29 (0)	1/29 (3.4)
		Moderate	0/50 (0)	0/52 (0)	1/29 (3.4)	0/29 (0)
		Severe	0/50 (0)	0/52 (0)	0/29 (0)	0/29 (0)
	2	Mild	0/50 (0)	0/52 (0)	0/28 (0)	1/29 (3.4)
		Moderate	0/50 (0)	0/52 (0)	0/28 (0)	0/29 (0)
		Severe	0/50 (0)	0/52 (0)	0/28 (0)	0/29 (0)
	3	Mild	0/50 (0)	0/52 (0)	0/28 (0)	0/29 (0)
		Moderate	0/50 (0)	0/52 (0)	0/28 (0)	0/29 (0)
		Severe	0/50 (0)	0/52 (0)	0/28 (0)	0/29 (0)
	4	Mild	0/50 (0)	0/52 (0)	0/29 (0)	0/29 (0)
		Moderate	0/50 (0)	0/52 (0)	0/29 (0)	0/29 (0)
		Severe	0/50 (0)	0/52 (0)	0/29 (0)	0/29 (0)
	5	Mild	1/50 (2)	0/51 (0)	0/29 (0)	0/29 (0)
		Moderate	0/50 (0)	0/51 (0)	0/29 (0)	0/29 (0)
		Severe	0/50 (0)	0/51 (0)	1/29 (3.4)	0/29 (0)
	6	Mild	0/49 (0)	0/51 (0)	0/29 (0)	0/29 (0)
		Moderate	0/49 (0)	0/51 (0)	0/29 (0)	0/29 (0)
		Severe	0/49 (0)	0/51 (0)	1/29 (3.4)	0/29 (0)
Bruising	0	Mild	0/50 (0)	6/52 (11.5)	1/29 (3.4)	1/29 (3.4)
		Moderate	0/50 (0)	1/52 (1.9)	0/29 (0)	0/29 (0)
		Severe	0/50 (0)	0/52 (0)	0/29 (0)	0/29 (0)
	1	Mild	2/50 (4)	4/52 (7.7)	0/29 (0)	2/29 (6.9)
		Moderate	0/50 (0)	0/52 (0)	0/29 (0)	0/29 (0)
		Severe	0/50 (0)	0/52 (0)	0/29 (0)	0/29 (0)
	2	Mild	0/50 (0)	4/52 (7.7)	0/28 (0)	0/29 (0)
		Moderate	0/50 (0)	0/52 (0)	0/28 (0)	0/29 (0)
		Severe	0/50 (0)	0/52 (0)	0/28 (0)	0/29 (0)

	3	Mild	0/48 (0)	3/51 (5.9)	0/28 (0)	0/29 (0)
		Moderate	0/48 (0)	0/51 (0)	0/28 (0)	0/29 (0)
		Severe	0/48 (0)	0/51 (0)	0/28 (0)	0/29 (0)
	4	Mild	0/49 (0)	1/52 (1.9)	0/29 (0)	1/29 (3.4)
		Moderate	0/49 (0)	0/52 (0)	0/29 (0)	0/29 (0)
		Severe	0/49 (0)	0/52 (0)	0/29 (0)	0/29 (0)
	5	Mild	0/49 (0)	0/51 (0)	0/29 (0)	0/28 (0)
		Moderate	0/49 (0)	0/51 (0)	0/29 (0)	0/28 (0)
		Severe	0/49 (0)	0/51 (0)	0/29 (0)	0/28 (0)
	6	Mild	0/49 (0)	0/51 (0)	0/29 (0)	0/28 (0)
		Moderate	0/49 (0)	0/51 (0)	0/29 (0)	0/28 (0)
		Severe	0/49 (0)	0/51 (0)	0/29 (0)	0/28 (0)
Cough	0	Mild	2/50 (4)	2/52 (3.8)	2/29 (6.9)	2/29 (6.9)
		Moderate	0/50 (0)	1/52 (1.9)	0/29 (0)	0/29 (0)
		Severe	0/50 (0)	0/52 (0)	0/29 (0)	0/29 (0)
	1	Mild	2/50 (4)	6/52 (11.5)	3/29 (10.3)	3/29 (10.3)
		Moderate	0/50 (0)	0/52 (0)	0/29 (0)	0/29 (0)
		Severe	0/50 (0)	0/52 (0)	0/29 (0)	0/29 (0)
	2	Mild	1/50 (2)	3/52 (5.8)	0/29 (0)	1/29 (3.4)
		Moderate	0/50 (0)	0/52 (0)	1/29 (3.4)	0/29 (0)
		Severe	0/50 (0)	0/52 (0)	0/29 (0)	0/29 (0)
	3	Mild	1/50 (2)	3/52 (5.8)	0/29 (0)	0/29 (0)
		Moderate	0/50 (0)	0/52 (0)	0/29 (0)	0/29 (0)
		Severe	0/50 (0)	0/52 (0)	0/29 (0)	0/29 (0)
	4	Mild	3/50 (6)	2/52 (3.8)	1/29 (3.4)	0/29 (0)
		Moderate	0/50 (0)	0/52 (0)	0/29 (0)	0/29 (0)
		Severe	0/50 (0)	0/52 (0)	0/29 (0)	0/29 (0)
	5	Mild	2/50 (4)	2/51 (3.9)	0/29 (0)	0/29 (0)
		Moderate	0/50 (0)	0/51 (0)	0/29 (0)	0/29 (0)
		Severe	0/50 (0)	0/51 (0)	0/29 (0)	0/29 (0)
	6	Mild	1/49 (2)	1/50 (2)	0/29 (0)	1/29 (3.4)
		Moderate	0/49 (0)	0/50 (0)	0/29 (0)	0/29 (0)
		Severe	0/49 (0)	0/50 (0)	0/29 (0)	0/29 (0)
Feverish	0	Yes	1/50 (2)	6/52 (11.5)	4/29 (13.8)	1/29 (3.4)
	1	Yes	1/49 (2)	6/52 (11.5)	1/26 (3.8)	3/29 (10.3)
	2	Yes	1/48 (2.1)	2/52 (3.8)	1/27 (3.7)	1/28 (3.6)
	3	Yes	2/48 (4.2)	3/50 (6)	1/28 (3.6)	1/28 (3.6)
	4	Yes	2/49 (4.1)	3/52 (5.8)	1/28 (3.6)	0/27 (0)
	5	Yes	2/49 (4.1)	3/49 (6.1)	1/27 (3.7)	0/27 (0)
	6	Yes	1/48 (2.1)	2/51 (3.9)	1/28 (3.6)	2/27 (7.4)
Hardness	0	Mild	0/50 (0)	6/52 (11.5)	2/29 (6.9)	1/29 (3.4)
		Moderate	0/50 (0)	4/52 (7.7)	0/29 (0)	0/29 (0)
		Severe	0/50 (0)	1/52 (1.9)	0/29 (0)	0/29 (0)
	1	Mild	1/50 (2)	10/52 (19.2)	0/29 (0)	4/29 (13.8)
		Moderate	0/50 (0)	2/52 (3.8)	0/29 (0)	0/29 (0)
		Severe	0/50 (0)	0/52 (0)	0/29 (0)	0/29 (0)
	2	Mild	0/50 (0)	8/52 (15.4)	0/28 (0)	0/29 (0)
		Moderate	0/50 (0)	0/52 (0)	0/28 (0)	1/29 (3.4)
		Severe	1/50 (2)	0/52 (0)	0/28 (0)	0/29 (0)
	3	Mild	0/48 (0)	6/52 (11.5)	1/29 (3.4)	1/29 (3.4)
		Moderate	0/48 (0)	1/52 (1.9)	0/29 (0)	0/29 (0)
		Severe	1/48 (2.1)	0/52 (0)	0/29 (0)	0/29 (0)
	4	Mild	0/49 (0)	3/52 (5.8)	0/29 (0)	2/29 (6.9)
		Moderate	0/49 (0)	2/52 (3.8)	0/29 (0)	0/29 (0)
		Severe	0/49 (0)	0/52 (0)	0/29 (0)	0/29 (0)
	5	Mild	0/49 (0)	3/51 (5.9)	0/29 (0)	0/28 (0)
		Moderate	0/49 (0)	0/51 (0)	0/29 (0)	0/28 (0)

		Severe	0/49 (0)	0/51 (0)	0/29 (0)	0/28 (0)
	6	Mild	0/49 (0)	3/51 (5.9)	0/29 (0)	0/28 (0)
		Moderate	0/49 (0)	0/51 (0)	0/29 (0)	0/28 (0)
		Severe	0/49 (0)	0/51 (0)	0/29 (0)	0/28 (0)
Headache	0	Mild	3/50 (6)	9/52 (17.3)	8/29 (27.6)	7/28 (25)
		Moderate	2/50 (4)	4/52 (7.7)	0/29 (0)	0/28 (0)
		Severe	0/50 (0)	0/52 (0)	0/29 (0)	0/28 (0)
	1	Mild	2/47 (4.3)	11/52 (21.2)	4/29 (13.8)	4/29 (13.8)
		Moderate	1/47 (2.1)	5/52 (9.6)	0/29 (0)	3/29 (10.3)
		Severe	0/47 (0)	0/52 (0)	0/29 (0)	0/29 (0)
	2	Mild	5/50 (10)	8/51 (15.7)	1/29 (3.4)	4/29 (13.8)
		Moderate	1/50 (2)	3/51 (5.9)	0/29 (0)	0/29 (0)
		Severe	0/50 (0)	0/51 (0)	0/29 (0)	0/29 (0)
	3	Mild	2/49 (4.1)	7/52 (13.5)	3/29 (10.3)	1/29 (3.4)
		Moderate	2/49 (4.1)	3/52 (5.8)	0/29 (0)	0/29 (0)
		Severe	1/49 (2)	0/52 (0)	0/29 (0)	0/29 (0)
	4	Mild	1/49 (2)	3/52 (5.8)	0/29 (0)	2/29 (6.9)
		Moderate	2/49 (4.1)	1/52 (1.9)	1/29 (3.4)	0/29 (0)
		Severe	0/49 (0)	0/52 (0)	0/29 (0)	0/29 (0)
	5	Mild	4/50 (8)	3/51 (5.9)	1/29 (3.4)	1/29 (3.4)
		Moderate	1/50 (2)	0/51 (0)	0/29 (0)	0/29 (0)
		Severe	0/50 (0)	0/51 (0)	1/29 (3.4)	0/29 (0)
	6	Mild	4/49 (8.2)	1/51 (2)	0/29 (0)	2/28 (7.1)
		Moderate	1/49 (2)	0/51 (0)	1/29 (3.4)	0/28 (0)
		Severe	0/49 (0)	0/51 (0)	0/29 (0)	0/28 (0)
Itching	0	Mild	3/50 (6)	6/52 (11.5)	2/29 (6.9)	6/29 (20.7)
		Moderate	0/50 (0)	3/52 (5.8)	0/29 (0)	0/29 (0)
		Severe	0/50 (0)	0/52 (0)	1/29 (3.4)	0/29 (0)
	1	Mild	4/50 (8)	4/52 (7.7)	2/29 (6.9)	4/29 (13.8)
		Moderate	0/50 (0)	2/52 (3.8)	0/29 (0)	0/29 (0)
		Severe	0/50 (0)	0/52 (0)	1/29 (3.4)	0/29 (0)
	2	Mild	1/50 (2)	5/52 (9.6)	0/28 (0)	2/29 (6.9)
		Moderate	1/50 (2)	0/52 (0)	1/28 (3.6)	0/29 (0)
		Severe	0/50 (0)	0/52 (0)	0/28 (0)	0/29 (0)
	3	Mild	0/49 (0)	5/52 (9.6)	0/29 (0)	2/29 (6.9)
		Moderate	0/49 (0)	0/52 (0)	1/29 (3.4)	0/29 (0)
		Severe	0/49 (0)	0/52 (0)	0/29 (0)	0/29 (0)
	4	Mild	1/49 (2)	3/51 (5.9)	0/29 (0)	2/29 (6.9)
		Moderate	0/49 (0)	1/51 (2)	0/29 (0)	0/29 (0)
		Severe	0/49 (0)	0/51 (0)	0/29 (0)	1/29 (3.4)
	5	Mild	0/49 (0)	1/51 (2)	0/29 (0)	0/28 (0)
		Moderate	1/49 (2)	0/51 (0)	0/29 (0)	1/28 (3.6)
		Severe	0/49 (0)	0/51 (0)	0/29 (0)	0/28 (0)
	6	Mild	0/49 (0)	1/51 (2)	0/29 (0)	0/28 (0)
		Moderate	1/49 (2)	0/51 (0)	0/29 (0)	1/28 (3.6)
		Severe	0/49 (0)	0/51 (0)	0/29 (0)	0/28 (0)
Joint pain	0	Mild	2/50 (4)	10/52 (19.2)	2/29 (6.9)	6/29 (20.7)
		Moderate	1/50 (2)	1/52 (1.9)	0/29 (0)	0/29 (0)
		Severe	0/50 (0)	0/52 (0)	0/29 (0)	0/29 (0)
	1	Mild	1/50 (2)	7/51 (13.7)	3/29 (10.3)	5/29 (17.2)
		Moderate	2/50 (4)	2/51 (3.9)	0/29 (0)	1/29 (3.4)
		Severe	0/50 (0)	0/51 (0)	0/29 (0)	0/29 (0)
	2	Mild	1/50 (2)	2/52 (3.8)	3/29 (10.3)	3/29 (10.3)
		Moderate	1/50 (2)	2/52 (3.8)	0/29 (0)	0/29 (0)
		Severe	0/50 (0)	1/52 (1.9)	0/29 (0)	0/29 (0)
	3	Mild	0/49 (0)	2/52 (3.8)	3/28 (10.7)	4/29 (13.8)
		Moderate	1/49 (2)	2/52 (3.8)	0/28 (0)	0/29 (0)

		Severe	1/49 (2)	0/52 (0)	0/28 (0)	0/29 (0)
	4	Mild	1/50 (2)	4/50 (8)	3/29 (10.3)	1/29 (3.4)
		Moderate	0/50 (0)	0/50 (0)	0/29 (0)	0/29 (0)
		Severe	0/50 (0)	0/50 (0)	0/29 (0)	0/29 (0)
	5	Mild	2/50 (4)	1/51 (2)	1/29 (3.4)	1/29 (3.4)
		Moderate	0/50 (0)	0/51 (0)	0/29 (0)	0/29 (0)
		Severe	0/50 (0)	0/51 (0)	0/29 (0)	0/29 (0)
	6	Mild	1/49 (2)	2/51 (3.9)	0/29 (0)	2/29 (6.9)
		Moderate	0/49 (0)	0/51 (0)	0/29 (0)	0/29 (0)
		Severe	0/49 (0)	0/51 (0)	0/29 (0)	0/29 (0)
Muscle pain	0	Mild	2/50 (4)	12/52 (23.1)	4/29 (13.8)	7/29 (24.1)
		Moderate	1/50 (2)	1/52 (1.9)	0/29 (0)	0/29 (0)
		Severe	0/50 (0)	0/52 (0)	0/29 (0)	0/29 (0)
	1	Mild	1/50 (2)	10/51 (19.6)	2/29 (6.9)	6/29 (20.7)
		Moderate	3/50 (6)	2/51 (3.9)	0/29 (0)	1/29 (3.4)
		Severe	0/50 (0)	0/51 (0)	0/29 (0)	0/29 (0)
	2	Mild	4/50 (8)	5/52 (9.6)	1/29 (3.4)	2/29 (6.9)
		Moderate	1/50 (2)	1/52 (1.9)	0/29 (0)	0/29 (0)
		Severe	0/50 (0)	1/52 (1.9)	0/29 (0)	0/29 (0)
	3	Mild	1/49 (2)	2/51 (3.9)	4/29 (13.8)	1/29 (3.4)
		Moderate	1/49 (2)	0/51 (0)	0/29 (0)	0/29 (0)
		Severe	1/49 (2)	1/51 (2)	0/29 (0)	0/29 (0)
	4	Mild	2/50 (4)	4/50 (8)	2/29 (6.9)	1/29 (3.4)
		Moderate	0/50 (0)	0/50 (0)	0/29 (0)	0/29 (0)
		Severe	0/50 (0)	0/50 (0)	0/29 (0)	0/29 (0)
	5	Mild	1/50 (2)	2/51 (3.9)	1/29 (3.4)	1/29 (3.4)
		Moderate	0/50 (0)	0/51 (0)	0/29 (0)	0/29 (0)
		Severe	0/50 (0)	0/51 (0)	0/29 (0)	0/29 (0)
	6	Mild	1/49 (2)	2/51 (3.9)	0/29 (0)	2/29 (6.9)
		Moderate	0/49 (0)	0/51 (0)	0/29 (0)	1/29 (3.4)
		Severe	0/49 (0)	0/51 (0)	0/29 (0)	0/29 (0)
Redness	0	Mild	0/49 (0)	1/48 (2.1)	0/28 (0)	0/29 (0)
		Moderate	0/49 (0)	0/48 (0)	0/28 (0)	0/29 (0)
		Severe	0/49 (0)	0/48 (0)	0/28 (0)	0/29 (0)
	1	Mild	0/50 (0)	1/49 (2)	0/28 (0)	0/29 (0)
		Moderate	0/50 (0)	0/49 (0)	0/28 (0)	0/29 (0)
		Severe	0/50 (0)	0/49 (0)	0/28 (0)	0/29 (0)
	2	Mild	0/50 (0)	1/49 (2)	0/28 (0)	0/29 (0)
		Moderate	0/50 (0)	0/49 (0)	0/28 (0)	0/29 (0)
		Severe	0/50 (0)	0/49 (0)	0/28 (0)	0/29 (0)
	3	Mild	0/49 (0)	0/49 (0)	0/28 (0)	1/29 (3.4)
		Moderate	0/49 (0)	0/49 (0)	0/28 (0)	0/29 (0)
		Severe	0/49 (0)	0/49 (0)	0/28 (0)	0/29 (0)
	4	Mild	0/49 (0)	0/49 (0)	0/28 (0)	1/29 (3.4)
		Moderate	0/49 (0)	0/49 (0)	0/28 (0)	0/29 (0)
		Severe	0/49 (0)	0/49 (0)	0/28 (0)	0/29 (0)
	5	Mild	0/49 (0)	0/48 (0)	0/28 (0)	1/29 (3.4)
		Moderate	0/49 (0)	0/48 (0)	0/28 (0)	0/29 (0)
		Severe	0/49 (0)	0/48 (0)	0/28 (0)	0/29 (0)
	6	Mild	0/47 (0)	0/48 (0)	0/28 (0)	0/29 (0)
		Moderate	0/47 (0)	0/48 (0)	0/28 (0)	0/29 (0)
		Severe	0/47 (0)	0/48 (0)	0/28 (0)	0/29 (0)
Rigors	0	Yes	0/50 (0)	2/52 (3.8)	1/29 (3.4)	0/29 (0)
	1	Yes	0/50 (0)	4/52 (7.7)	0/28 (0)	1/29 (3.4)
	2	Yes	0/50 (0)	3/52 (5.8)	0/29 (0)	0/29 (0)
	3	Yes	0/50 (0)	2/51 (3.9)	0/29 (0)	1/29 (3.4)
	4	Yes	0/49 (0)	1/52 (1.9)	0/28 (0)	0/29 (0)

	5	Yes	0/50 (0)	1/51 (2)	0/29 (0)	0/29 (0)
	6	Yes	0/48 (0)	1/51 (2)	0/29 (0)	0/29 (0)
SOB	0	Mild	0/50 (0)	3/52 (5.8)	0/29 (0)	1/29 (3.4)
		Moderate	0/50 (0)	0/52 (0)	0/29 (0)	0/29 (0)
		Severe	0/50 (0)	0/52 (0)	0/29 (0)	0/29 (0)
	1	Mild	1/50 (2)	2/52 (3.8)	0/29 (0)	1/29 (3.4)
		Moderate	0/50 (0)	0/52 (0)	0/29 (0)	0/29 (0)
		Severe	0/50 (0)	0/52 (0)	0/29 (0)	0/29 (0)
	2	Mild	0/50 (0)	1/52 (1.9)	0/29 (0)	0/29 (0)
		Moderate	0/50 (0)	0/52 (0)	0/29 (0)	1/29 (3.4)
		Severe	0/50 (0)	0/52 (0)	0/29 (0)	0/29 (0)
	3	Mild	0/50 (0)	1/52 (1.9)	0/28 (0)	0/29 (0)
		Moderate	0/50 (0)	0/52 (0)	0/28 (0)	0/29 (0)
		Severe	0/50 (0)	0/52 (0)	0/28 (0)	0/29 (0)
	4	Mild	0/50 (0)	1/52 (1.9)	0/29 (0)	0/29 (0)
		Moderate	0/50 (0)	0/52 (0)	0/29 (0)	0/29 (0)
		Severe	1/50 (2)	0/52 (0)	0/29 (0)	0/29 (0)
	5	Mild	0/50 (0)	0/51 (0)	0/29 (0)	0/29 (0)
		Moderate	0/50 (0)	0/51 (0)	0/29 (0)	0/29 (0)
		Severe	0/50 (0)	0/51 (0)	0/29 (0)	0/29 (0)
	6	Mild	0/49 (0)	0/51 (0)	0/29 (0)	0/29 (0)
		Moderate	0/49 (0)	0/51 (0)	0/29 (0)	0/29 (0)
		Severe	0/49 (0)	0/51 (0)	0/29 (0)	0/29 (0)
Sweating	0	Mild	0/50 (0)	4/51 (7.8)	4/29 (13.8)	2/29 (6.9)
		Moderate	0/50 (0)	0/51 (0)	0/29 (0)	0/29 (0)
		Severe	0/50 (0)	0/51 (0)	0/29 (0)	0/29 (0)
	1	Mild	0/50 (0)	1/52 (1.9)	0/28 (0)	0/29 (0)
		Moderate	0/50 (0)	2/52 (3.8)	0/28 (0)	2/29 (6.9)
		Severe	0/50 (0)	0/52 (0)	0/28 (0)	0/29 (0)
	2	Mild	0/50 (0)	3/52 (5.8)	0/28 (0)	1/29 (3.4)
		Moderate	0/50 (0)	0/52 (0)	0/28 (0)	1/29 (3.4)
		Severe	0/50 (0)	0/52 (0)	0/28 (0)	0/29 (0)
	3	Mild	1/50 (2)	3/52 (5.8)	0/29 (0)	0/29 (0)
		Moderate	0/50 (0)	0/52 (0)	0/29 (0)	0/29 (0)
		Severe	0/50 (0)	0/52 (0)	0/29 (0)	0/29 (0)
	4	Mild	0/50 (0)	1/52 (1.9)	0/29 (0)	1/29 (3.4)
		Moderate	0/50 (0)	0/52 (0)	0/29 (0)	0/29 (0)
		Severe	0/50 (0)	0/52 (0)	0/29 (0)	0/29 (0)
	5	Mild	1/50 (2)	0/51 (0)	1/29 (3.4)	0/29 (0)
		Moderate	0/50 (0)	0/51 (0)	0/29 (0)	0/29 (0)
		Severe	0/50 (0)	0/51 (0)	0/29 (0)	0/29 (0)
	6	Mild	1/49 (2)	0/51 (0)	0/29 (0)	0/29 (0)
		Moderate	0/49 (0)	0/51 (0)	0/29 (0)	0/29 (0)
		Severe	0/49 (0)	0/51 (0)	0/29 (0)	0/29 (0)
Swelling	0	Mild	0/49 (0)	1/47 (2.1)	0/28 (0)	0/29 (0)
		Moderate	0/49 (0)	0/47 (0)	0/28 (0)	0/29 (0)
		Severe	0/49 (0)	0/47 (0)	0/28 (0)	0/29 (0)
	1	Mild	0/50 (0)	1/49 (2)	0/28 (0)	0/29 (0)
		Moderate	0/50 (0)	0/49 (0)	0/28 (0)	0/29 (0)
		Severe	0/50 (0)	0/49 (0)	0/28 (0)	0/29 (0)
	2	Mild	0/50 (0)	1/48 (2.1)	0/28 (0)	0/29 (0)
		Moderate	0/50 (0)	0/48 (0)	0/28 (0)	0/29 (0)
		Severe	0/50 (0)	0/48 (0)	0/28 (0)	0/29 (0)
	3	Mild	0/49 (0)	1/48 (2.1)	0/28 (0)	1/29 (3.4)
		Moderate	0/49 (0)	0/48 (0)	0/28 (0)	0/29 (0)
		Severe	0/49 (0)	0/48 (0)	0/28 (0)	0/29 (0)
	4	Mild	0/49 (0)	1/49 (2)	0/28 (0)	1/29 (3.4)

		Moderate	0/49 (0)	0/49 (0)	0/28 (0)	0/29 (0)
		Severe	0/49 (0)	0/49 (0)	0/28 (0)	0/29 (0)
	5	Mild	0/49 (0)	0/47 (0)	0/28 (0)	1/29 (3.4)
		Moderate	0/49 (0)	0/47 (0)	0/28 (0)	0/29 (0)
		Severe	0/49 (0)	0/47 (0)	0/28 (0)	0/29 (0)
	6	Mild	0/47 (0)	0/47 (0)	0/28 (0)	0/29 (0)
		Moderate	0/47 (0)	0/47 (0)	0/28 (0)	0/29 (0)
		Severe	0/47 (0)	0/47 (0)	0/28 (0)	0/29 (0)
Temperature	0	Mild	0/48 (0)	0/52 (0)	0/29 (0)	0/29 (0)
		Moderate	0/48 (0)	0/52 (0)	0/29 (0)	0/29 (0)
		Severe	0/48 (0)	0/52 (0)	0/29 (0)	0/29 (0)
		Grade 4	0/48 (0)	0/52 (0)	0/29 (0)	0/29 (0)
	**1**	Mild	0/50 (0)	0/52 (0)	0/29 (0)	1/29 (3.4)
		Moderate	0/50 (0)	0/52 (0)	0/29 (0)	0/29 (0)
		Severe	0/50 (0)	0/52 (0)	1/29 (3.4)	0/29 (0)
		Grade 4	0/50 (0)	0/52 (0)	0/29 (0)	0/29 (0)
	**2**	Mild	0/50 (0)	0/52 (0)	0/29 (0)	0/29 (0)
		Moderate	0/50 (0)	0/52 (0)	0/29 (0)	0/29 (0)
		Severe	0/50 (0)	0/52 (0)	0/29 (0)	0/29 (0)
		Grade 4	0/50 (0)	0/52 (0)	0/29 (0)	1/29 (3.4)
	3	Mild	0/50 (0)	0/52 (0)	0/29 (0)	0/29 (0)
		Moderate	0/50 (0)	0/52 (0)	0/29 (0)	0/29 (0)
		Severe	0/50 (0)	0/52 (0)	0/29 (0)	0/29 (0)
		Grade 4	0/50 (0)	0/52 (0)	0/29 (0)	0/29 (0)
	4	Mild	0/50 (0)	0/52 (0)	0/29 (0)	0/28 (0)
		Moderate	0/50 (0)	0/52 (0)	0/29 (0)	0/28 (0)
		Severe	0/50 (0)	0/52 (0)	0/29 (0)	0/28 (0)
		Grade 4	0/50 (0)	0/52 (0)	0/29 (0)	0/28 (0)
	5	Mild	0/50 (0)	0/51 (0)	0/29 (0)	0/29 (0)
		Moderate	0/50 (0)	0/51 (0)	0/29 (0)	0/29 (0)
		Severe	0/50 (0)	0/51 (0)	0/29 (0)	0/29 (0)
		Grade 4	0/50 (0)	0/51 (0)	0/29 (0)	0/29 (0)
	6	Mild	0/44 (0)	0/50 (0)	0/29 (0)	0/29 (0)
		Moderate	0/44 (0)	0/50 (0)	0/29 (0)	1/29 (3.4)
		Severe	0/44 (0)	0/50 (0)	0/29 (0)	0/29 (0)
		Grade 4	0/44 (0)	0/50 (0)	0/29 (0)	0/29 (0)
Tenderness	0	Mild	0/50 (0)	3/52 (5.8)	3/29 (10.3)	5/29 (17.2)
		Moderate	0/50 (0)	5/52 (9.6)	0/29 (0)	0/29 (0)
		Severe	0/50 (0)	1/52 (1.9)	0/29 (0)	0/29 (0)
	1	Mild	1/50 (2)	11/52 (21.2)	2/29 (6.9)	3/29 (10.3)
		Moderate	0/50 (0)	5/52 (9.6)	0/29 (0)	1/29 (3.4)
		Severe	0/50 (0)	0/52 (0)	0/29 (0)	0/29 (0)
	2	Mild	0/50 (0)	6/51 (11.8)	0/29 (0)	2/29 (6.9)
		Moderate	0/50 (0)	0/51 (0)	0/29 (0)	0/29 (0)
		Severe	1/50 (2)	0/51 (0)	0/29 (0)	0/29 (0)
	3	Mild	0/49 (0)	5/52 (9.6)	1/29 (3.4)	3/29 (10.3)
		Moderate	0/49 (0)	1/52 (1.9)	1/29 (3.4)	0/29 (0)
		Severe	0/49 (0)	0/52 (0)	0/29 (0)	0/29 (0)
	4	Mild	0/49 (0)	2/52 (3.8)	0/29 (0)	1/28 (3.6)
		Moderate	0/49 (0)	1/52 (1.9)	0/29 (0)	0/28 (0)
		Severe	0/49 (0)	0/52 (0)	0/29 (0)	0/28 (0)
	5	Mild	0/49 (0)	2/51 (3.9)	0/29 (0)	0/28 (0)
		Moderate	0/49 (0)	0/51 (0)	0/29 (0)	0/28 (0)
		Severe	0/49 (0)	0/51 (0)	0/29 (0)	0/28 (0)
	6	Mild	0/49 (0)	2/51 (3.9)	0/29 (0)	0/28 (0)
		Moderate	0/49 (0)	0/51 (0)	0/29 (0)	0/28 (0)
		Severe	0/49 (0)	0/51 (0)	0/29 (0)	0/28 (0)

Weakness	0	Mild	3/50 (6)	6/52 (11.5)	3/29 (10.3)	6/29 (20.7)
		Moderate	1/50 (2)	3/52 (5.8)	1/29 (3.4)	0/29 (0)
		Severe	0/50 (0)	0/52 (0)	0/29 (0)	0/29 (0)
	1	Mild	4/50 (8)	8/52 (15.4)	2/29 (6.9)	4/29 (13.8)
		Moderate	1/50 (2)	3/52 (5.8)	1/29 (3.4)	2/29 (6.9)
		Severe	0/50 (0)	0/52 (0)	0/29 (0)	0/29 (0)
	2	Mild	3/50 (6)	6/52 (11.5)	1/28 (3.6)	4/29 (13.8)
		Moderate	1/50 (2)	2/52 (3.8)	0/28 (0)	0/29 (0)
		Severe	0/50 (0)	0/52 (0)	0/28 (0)	0/29 (0)
	3	Mild	2/50 (4)	4/52 (7.7)	2/29 (6.9)	1/29 (3.4)
		Moderate	1/50 (2)	1/52 (1.9)	0/29 (0)	0/29 (0)
		Severe	0/50 (0)	0/52 (0)	0/29 (0)	0/29 (0)
	4	Mild	2/50 (4)	0/52 (0)	0/29 (0)	4/29 (13.8)
		Moderate	1/50 (2)	0/52 (0)	0/29 (0)	0/29 (0)
		Severe	0/50 (0)	0/52 (0)	0/29 (0)	0/29 (0)
	5	Mild	4/50 (8)	1/51 (2)	0/29 (0)	1/29 (3.4)
		Moderate	1/50 (2)	0/51 (0)	0/29 (0)	0/29 (0)
		Severe	0/50 (0)	0/51 (0)	0/29 (0)	0/29 (0)
	6	Mild	2/49 (4.1)	1/51 (2)	0/29 (0)	1/29 (3.4)
		Moderate	2/49 (4.1)	0/51 (0)	0/29 (0)	0/29 (0)
		Severe	0/49 (0)	0/51 (0)	0/29 (0)	0/29 (0)
**After booster dose**						
Ageusia	0	Mild	0/50 (0)	1/52 (1.9)	1/28 (3.6)	0/29 (0)
		Moderate	0/50 (0)	0/52 (0)	0/28 (0)	0/29 (0)
		Severe	0/50 (0)	0/52 (0)	1/28 (3.6)	0/29 (0)
	1	Mild	0/50 (0)	0/52 (0)	1/28 (3.6)	2/29 (6.9)
		Moderate	0/50 (0)	0/52 (0)	0/28 (0)	0/29 (0)
		Severe	0/50 (0)	0/52 (0)	1/28 (3.6)	0/29 (0)
	2	Mild	0/50 (0)	1/52 (1.9)	1/28 (3.6)	2/29 (6.9)
		Moderate	0/50 (0)	0/52 (0)	0/28 (0)	0/29 (0)
		Severe	0/50 (0)	0/52 (0)	1/28 (3.6)	0/29 (0)
	3	Mild	1/50 (2)	0/52 (0)	1/28 (3.6)	0/29 (0)
		Moderate	0/50 (0)	0/52 (0)	0/28 (0)	0/29 (0)
		Severe	0/50 (0)	0/52 (0)	1/28 (3.6)	0/29 (0)
	4	Mild	1/50 (2)	0/51 (0)	1/28 (3.6)	0/29 (0)
		Moderate	0/50 (0)	0/51 (0)	0/28 (0)	0/29 (0)
		Severe	0/50 (0)	0/51 (0)	1/28 (3.6)	0/29 (0)
	5	Mild	1/50 (2)	1/51 (2)	1/28 (3.6)	0/29 (0)
		Moderate	0/50 (0)	0/51 (0)	0/28 (0)	0/29 (0)
		Severe	0/50 (0)	0/51 (0)	1/28 (3.6)	0/29 (0)
	6	Mild	0/49 (0)	1/50 (2)	1/28 (3.6)	0/29 (0)
		Moderate	0/49 (0)	0/50 (0)	0/28 (0)	0/29 (0)
		Severe	0/49 (0)	0/50 (0)	2/28 (7.1)	0/29 (0)
Anosmia	0	Mild	0/50 (0)	1/52 (1.9)	1/28 (3.6)	0/29 (0)
		Moderate	0/50 (0)	0/52 (0)	0/28 (0)	0/29 (0)
		Severe	0/50 (0)	0/52 (0)	2/28 (7.1)	0/29 (0)
	1	Mild	1/50 (2)	0/52 (0)	1/28 (3.6)	2/29 (6.9)
		Moderate	0/50 (0)	0/52 (0)	0/28 (0)	0/29 (0)
		Severe	0/50 (0)	0/52 (0)	2/28 (7.1)	0/29 (0)
	2	Mild	1/50 (2)	1/52 (1.9)	1/28 (3.6)	2/29 (6.9)
		Moderate	0/50 (0)	0/52 (0)	0/28 (0)	0/29 (0)
		Severe	0/50 (0)	0/52 (0)	2/28 (7.1)	0/29 (0)
	3	Mild	1/50 (2)	0/52 (0)	1/28 (3.6)	1/29 (3.4)
		Moderate	0/50 (0)	0/52 (0)	0/28 (0)	0/29 (0)
		Severe	0/50 (0)	0/52 (0)	2/28 (7.1)	0/29 (0)
	4	Mild	0/50 (0)	0/51 (0)	1/28 (3.6)	1/29 (3.4)
		Moderate	0/50 (0)	0/51 (0)	0/28 (0)	0/29 (0)

		Severe	0/50 (0)	0/51 (0)	2/28 (7.1)	0/29 (0)
	5	Mild	1/50 (2)	0/51 (0)	1/27 (3.7)	0/29 (0)
		Moderate	0/50 (0)	0/51 (0)	0/27 (0)	0/29 (0)
		Severe	0/50 (0)	0/51 (0)	1/27 (3.7)	0/29 (0)
	6	Mild	0/50 (0)	0/50 (0)	1/28 (3.6)	0/29 (0)
		Moderate	0/50 (0)	0/50 (0)	0/28 (0)	0/29 (0)
		Severe	0/50 (0)	0/50 (0)	2/28 (7.1)	0/29 (0)
Bruising	0	Mild	1/50 (2)	3/52 (5.8)	0/28 (0)	1/29 (3.4)
		Moderate	0/50 (0)	1/52 (1.9)	0/28 (0)	1/29 (3.4)
		Severe	0/50 (0)	0/52 (0)	0/28 (0)	0/29 (0)
	1	Mild	0/50 (0)	5/52 (9.6)	0/28 (0)	0/29 (0)
		Moderate	2/50 (4)	1/52 (1.9)	0/28 (0)	1/29 (3.4)
		Severe	0/50 (0)	0/52 (0)	0/28 (0)	0/29 (0)
	2	Mild	0/50 (0)	3/52 (5.8)	0/28 (0)	1/29 (3.4)
		Moderate	0/50 (0)	0/52 (0)	0/28 (0)	0/29 (0)
		Severe	0/50 (0)	0/52 (0)	0/28 (0)	0/29 (0)
	3	Mild	0/50 (0)	2/52 (3.8)	0/28 (0)	0/29 (0)
		Moderate	0/50 (0)	0/52 (0)	0/28 (0)	0/29 (0)
		Severe	0/50 (0)	0/52 (0)	0/28 (0)	0/29 (0)
	4	Mild	0/50 (0)	1/51 (2)	0/27 (0)	0/29 (0)
		Moderate	0/50 (0)	0/51 (0)	0/27 (0)	0/29 (0)
		Severe	0/50 (0)	0/51 (0)	0/27 (0)	0/29 (0)
	5	Mild	0/50 (0)	0/51 (0)	0/27 (0)	0/29 (0)
		Moderate	0/50 (0)	0/51 (0)	0/27 (0)	0/29 (0)
		Severe	0/50 (0)	0/51 (0)	0/27 (0)	0/29 (0)
	6	Mild	0/50 (0)	0/51 (0)	0/27 (0)	0/29 (0)
		Moderate	0/50 (0)	0/51 (0)	0/27 (0)	0/29 (0)
		Severe	0/50 (0)	0/51 (0)	0/27 (0)	0/29 (0)
Cough	0	Mild	0/50 (0)	4/52 (7.7)	0/28 (0)	3/29 (10.3)
		Moderate	1/50 (2)	0/52 (0)	0/28 (0)	0/29 (0)
		Severe	0/50 (0)	0/52 (0)	0/28 (0)	0/29 (0)
	1	Mild	0/50 (0)	3/52 (5.8)	1/28 (3.6)	1/29 (3.4)
		Moderate	0/50 (0)	0/52 (0)	0/28 (0)	1/29 (3.4)
		Severe	0/50 (0)	0/52 (0)	0/28 (0)	0/29 (0)
	2	Mild	0/50 (0)	2/52 (3.8)	0/28 (0)	2/29 (6.9)
		Moderate	0/50 (0)	0/52 (0)	0/28 (0)	0/29 (0)
		Severe	0/50 (0)	0/52 (0)	0/28 (0)	0/29 (0)
	3	Mild	0/50 (0)	2/52 (3.8)	0/28 (0)	1/29 (3.4)
		Moderate	0/50 (0)	0/52 (0)	0/28 (0)	0/29 (0)
		Severe	0/50 (0)	0/52 (0)	0/28 (0)	0/29 (0)
	4	Mild	1/50 (2)	1/51 (2)	0/28 (0)	1/29 (3.4)
		Moderate	0/50 (0)	0/51 (0)	0/28 (0)	0/29 (0)
		Severe	0/50 (0)	0/51 (0)	0/28 (0)	0/29 (0)
	5	Mild	1/50 (2)	1/51 (2)	1/28 (3.6)	0/29 (0)
		Moderate	0/50 (0)	0/51 (0)	0/28 (0)	0/29 (0)
		Severe	0/50 (0)	0/51 (0)	0/28 (0)	0/29 (0)
	6	Mild	1/50 (2)	1/50 (2)	1/28 (3.6)	0/29 (0)
		Moderate	0/50 (0)	0/50 (0)	0/28 (0)	0/29 (0)
		Severe	0/50 (0)	0/50 (0)	0/28 (0)	0/29 (0)
Feverish	0	Yes	1/50 (2)	5/49 (10.2)	1/27 (3.7)	1/29 (3.4)
	1	Yes	1/50 (2)	5/45 (11.1)	0/26 (0)	1/29 (3.4)
	2	Yes	1/50 (2)	3/48 (6.2)	1/26 (3.8)	2/29 (6.9)
	3	Yes	0/50 (0)	3/47 (6.4)	0/26 (0)	1/28 (3.6)
	4	Yes	0/49 (0)	3/46 (6.5)	0/26 (0)	1/29 (3.4)
	5	Yes	0/48 (0)	4/46 (8.7)	0/26 (0)	0/27 (0)
	6	Yes	0/49 (0)	4/47 (8.5)	1/26 (3.8)	0/28 (0)
Hardness	0	Mild	2/50 (4)	8/52 (15.4)	0/28 (0)	4/29 (13.8)

		Moderate	1/50 (2)	2/52 (3.8)	0/28 (0)	1/29 (3.4)
		Severe	0/50 (0)	1/52 (1.9)	0/28 (0)	0/29 (0)
	1	Mild	2/50 (4)	6/52 (11.5)	0/28 (0)	1/29 (3.4)
		Moderate	1/50 (2)	1/52 (1.9)	0/28 (0)	1/29 (3.4)
		Severe	0/50 (0)	1/52 (1.9)	0/28 (0)	0/29 (0)
	2	Mild	1/50 (2)	3/52 (5.8)	0/28 (0)	2/29 (6.9)
		Moderate	0/50 (0)	2/52 (3.8)	0/28 (0)	0/29 (0)
		Severe	0/50 (0)	0/52 (0)	0/28 (0)	0/29 (0)
	3	Mild	1/50 (2)	3/52 (5.8)	0/28 (0)	0/29 (0)
		Moderate	0/50 (0)	0/52 (0)	0/28 (0)	0/29 (0)
		Severe	0/50 (0)	0/52 (0)	0/28 (0)	0/29 (0)
	4	Mild	0/50 (0)	1/51 (2)	0/27 (0)	0/29 (0)
		Moderate	0/50 (0)	0/51 (0)	0/27 (0)	0/29 (0)
		Severe	0/50 (0)	0/51 (0)	0/27 (0)	0/29 (0)
	5	Mild	0/50 (0)	1/51 (2)	0/27 (0)	0/29 (0)
		Moderate	0/50 (0)	0/51 (0)	0/27 (0)	0/29 (0)
		Severe	0/50 (0)	0/51 (0)	0/27 (0)	0/29 (0)
	6	Mild	0/50 (0)	0/51 (0)	0/27 (0)	0/29 (0)
		Moderate	0/50 (0)	0/51 (0)	0/27 (0)	0/29 (0)
		Severe	0/50 (0)	0/51 (0)	0/27 (0)	0/29 (0)
Headache	0	Mild	6/50 (12)	8/52 (15.4)	2/28 (7.1)	6/29 (20.7)
		Moderate	0/50 (0)	5/52 (9.6)	0/28 (0)	0/29 (0)
		Severe	0/50 (0)	0/52 (0)	1/28 (3.6)	0/29 (0)
	1	Mild	5/50 (10)	3/52 (5.8)	3/28 (10.7)	4/29 (13.8)
		Moderate	0/50 (0)	5/52 (9.6)	1/28 (3.6)	1/29 (3.4)
		Severe	0/50 (0)	0/52 (0)	0/28 (0)	0/29 (0)
	2	Mild	1/50 (2)	5/52 (9.6)	1/28 (3.6)	3/29 (10.3)
		Moderate	0/50 (0)	5/52 (9.6)	0/28 (0)	1/29 (3.4)
		Severe	0/50 (0)	0/52 (0)	0/28 (0)	0/29 (0)
	3	Mild	2/50 (4)	4/52 (7.7)	2/28 (7.1)	2/29 (6.9)
		Moderate	1/50 (2)	2/52 (3.8)	0/28 (0)	1/29 (3.4)
		Severe	0/50 (0)	0/52 (0)	0/28 (0)	0/29 (0)
	4	Mild	1/50 (2)	3/51 (5.9)	1/27 (3.7)	2/29 (6.9)
		Moderate	1/50 (2)	0/51 (0)	1/27 (3.7)	0/29 (0)
		Severe	0/50 (0)	0/51 (0)	0/27 (0)	0/29 (0)
	5	Mild	2/50 (4)	6/51 (11.8)	1/28 (3.6)	1/28 (3.6)
		Moderate	1/50 (2)	0/51 (0)	0/28 (0)	0/28 (0)
		Severe	0/50 (0)	0/51 (0)	0/28 (0)	0/28 (0)
	6	Mild	3/50 (6)	5/49 (10.2)	1/28 (3.6)	0/29 (0)
		Moderate	0/50 (0)	0/49 (0)	0/28 (0)	0/29 (0)
		Severe	0/50 (0)	0/49 (0)	0/28 (0)	0/29 (0)
Itching	0	Mild	1/50 (2)	11/52 (21.2)	1/28 (3.6)	5/29 (17.2)
		Moderate	2/50 (4)	0/52 (0)	0/28 (0)	0/29 (0)
		Severe	0/50 (0)	0/52 (0)	0/28 (0)	0/29 (0)
	1	Mild	0/50 (0)	5/52 (9.6)	0/28 (0)	2/29 (6.9)
		Moderate	3/50 (6)	0/52 (0)	0/28 (0)	0/29 (0)
		Severe	0/50 (0)	0/52 (0)	0/28 (0)	0/29 (0)
	2	Mild	1/50 (2)	3/52 (5.8)	0/28 (0)	1/29 (3.4)
		Moderate	1/50 (2)	0/52 (0)	0/28 (0)	0/29 (0)
		Severe	0/50 (0)	0/52 (0)	0/28 (0)	0/29 (0)
	3	Mild	0/50 (0)	2/52 (3.8)	0/28 (0)	1/29 (3.4)
		Moderate	1/50 (2)	0/52 (0)	0/28 (0)	0/29 (0)
		Severe	0/50 (0)	0/52 (0)	0/28 (0)	0/29 (0)
	4	Mild	0/50 (0)	1/51 (2)	0/27 (0)	1/29 (3.4)
		Moderate	1/50 (2)	0/51 (0)	0/27 (0)	0/29 (0)
		Severe	0/50 (0)	0/51 (0)	0/27 (0)	0/29 (0)
	5	Mild	0/50 (0)	1/51 (2)	0/27 (0)	0/29 (0)

		Moderate	1/50 (2)	0/51 (0)	0/27 (0)	0/29 (0)
		Severe	0/50 (0)	0/51 (0)	0/27 (0)	0/29 (0)
	6	Mild	1/50 (2)	0/51 (0)	0/27 (0)	0/28 (0)
		Moderate	1/50 (2)	0/51 (0)	0/27 (0)	0/28 (0)
		Severe	0/50 (0)	0/51 (0)	0/27 (0)	0/28 (0)
Joint pain	0	Mild	2/50 (4)	4/51 (7.8)	0/27 (0)	2/29 (6.9)
		Moderate	1/50 (2)	1/51 (2)	0/27 (0)	0/29 (0)
		Severe	0/50 (0)	0/51 (0)	0/27 (0)	0/29 (0)
	1	Mild	2/50 (4)	5/52 (9.6)	0/27 (0)	1/29 (3.4)
		Moderate	1/50 (2)	1/52 (1.9)	0/27 (0)	0/29 (0)
		Severe	0/50 (0)	0/52 (0)	0/27 (0)	0/29 (0)
	2	Mild	1/50 (2)	5/52 (9.6)	0/27 (0)	1/29 (3.4)
		Moderate	0/50 (0)	0/52 (0)	0/27 (0)	0/29 (0)
		Severe	0/50 (0)	0/52 (0)	0/27 (0)	0/29 (0)
	3	Mild	1/50 (2)	2/52 (3.8)	1/26 (3.8)	0/29 (0)
		Moderate	0/50 (0)	0/52 (0)	1/26 (3.8)	0/29 (0)
		Severe	0/50 (0)	0/52 (0)	0/26 (0)	0/29 (0)
	4	Mild	0/50 (0)	2/51 (3.9)	1/27 (3.7)	0/29 (0)
		Moderate	0/50 (0)	0/51 (0)	0/27 (0)	0/29 (0)
		Severe	0/50 (0)	0/51 (0)	0/27 (0)	0/29 (0)
	5	Mild	0/50 (0)	3/51 (5.9)	1/28 (3.6)	0/29 (0)
		Moderate	0/50 (0)	0/51 (0)	0/28 (0)	0/29 (0)
		Severe	0/50 (0)	0/51 (0)	0/28 (0)	0/29 (0)
	6	Mild	0/49 (0)	2/50 (4)	1/28 (3.6)	1/29 (3.4)
		Moderate	0/49 (0)	0/50 (0)	0/28 (0)	0/29 (0)
		Severe	0/49 (0)	0/50 (0)	0/28 (0)	0/29 (0)
Muscle pain	0	Mild	3/50 (6)	8/51 (15.7)	1/27 (3.7)	2/29 (6.9)
		Moderate	1/50 (2)	2/51 (3.9)	1/27 (3.7)	0/29 (0)
		Severe	0/50 (0)	0/51 (0)	0/27 (0)	0/29 (0)
	1	Mild	3/50 (6)	5/52 (9.6)	0/27 (0)	4/29 (13.8)
		Moderate	0/50 (0)	2/52 (3.8)	1/27 (3.7)	0/29 (0)
		Severe	0/50 (0)	0/52 (0)	0/27 (0)	0/29 (0)
	2	Mild	0/50 (0)	3/52 (5.8)	0/27 (0)	3/29 (10.3)
		Moderate	0/50 (0)	1/52 (1.9)	0/27 (0)	0/29 (0)
		Severe	0/50 (0)	0/52 (0)	0/27 (0)	0/29 (0)
	3	Mild	0/50 (0)	3/52 (5.8)	0/27 (0)	2/29 (6.9)
		Moderate	0/50 (0)	0/52 (0)	1/27 (3.7)	0/29 (0)
		Severe	0/50 (0)	0/52 (0)	0/27 (0)	0/29 (0)
	4	Mild	0/50 (0)	3/51 (5.9)	1/27 (3.7)	2/29 (6.9)
		Moderate	0/50 (0)	0/51 (0)	0/27 (0)	0/29 (0)
		Severe	0/50 (0)	0/51 (0)	0/27 (0)	0/29 (0)
	5	Mild	0/50 (0)	4/51 (7.8)	1/28 (3.6)	2/29 (6.9)
		Moderate	0/50 (0)	0/51 (0)	0/28 (0)	0/29 (0)
		Severe	0/50 (0)	0/51 (0)	0/28 (0)	0/29 (0)
	6	Mild	0/49 (0)	3/50 (6)	1/28 (3.6)	2/29 (6.9)
		Moderate	0/49 (0)	0/50 (0)	0/28 (0)	0/29 (0)
		Severe	0/49 (0)	0/50 (0)	0/28 (0)	0/29 (0)
Redness	0	Mild	0/49 (0)	1/51 (2)	0/27 (0)	1/29 (3.4)
		Moderate	0/49 (0)	0/51 (0)	0/27 (0)	0/29 (0)
		Severe	0/49 (0)	0/51 (0)	0/27 (0)	0/29 (0)
	1	Mild	0/49 (0)	1/51 (2)	0/27 (0)	1/29 (3.4)
		Moderate	0/49 (0)	0/51 (0)	0/27 (0)	0/29 (0)
		Severe	0/49 (0)	0/51 (0)	0/27 (0)	0/29 (0)
	2	Mild	0/49 (0)	1/52 (1.9)	0/27 (0)	1/29 (3.4)
		Moderate	0/49 (0)	0/52 (0)	0/27 (0)	0/29 (0)
		Severe	0/49 (0)	0/52 (0)	0/27 (0)	0/29 (0)
	3	Mild	0/49 (0)	1/52 (1.9)	0/27 (0)	0/29 (0)

		Moderate	0/49 (0)	0/52 (0)	0/27 (0)	0/29 (0)
		Severe	0/49 (0)	0/52 (0)	0/27 (0)	0/29 (0)
	4	Mild	0/49 (0)	0/51 (0)	0/27 (0)	0/29 (0)
		Moderate	0/49 (0)	0/51 (0)	0/27 (0)	0/29 (0)
		Severe	0/49 (0)	0/51 (0)	0/27 (0)	0/29 (0)
	5	Mild	0/48 (0)	0/51 (0)	0/26 (0)	0/29 (0)
		Moderate	0/48 (0)	0/51 (0)	0/26 (0)	0/29 (0)
		Severe	0/48 (0)	0/51 (0)	0/26 (0)	0/29 (0)
	6	Mild	0/47 (0)	0/51 (0)	0/26 (0)	0/29 (0)
		Moderate	0/47 (0)	0/51 (0)	0/26 (0)	0/29 (0)
		Severe	0/47 (0)	0/51 (0)	0/26 (0)	0/29 (0)
Rigors	0	Yes	0/50 (0)	4/52 (7.7)	1/28 (3.6)	0/29 (0)
	1	Yes	0/50 (0)	3/52 (5.8)	1/28 (3.6)	1/28 (3.6)
	2	Yes	0/50 (0)	3/52 (5.8)	0/28 (0)	2/29 (6.9)
	3	Yes	0/50 (0)	2/51 (3.9)	0/28 (0)	1/28 (3.6)
	4	Yes	0/50 (0)	2/51 (3.9)	0/28 (0)	0/28 (0)
	5	Yes	0/50 (0)	2/51 (3.9)	0/28 (0)	0/28 (0)
	6	Yes	0/50 (0)	1/50 (2)	0/28 (0)	0/28 (0)
SOB	0	Mild	0/50 (0)	1/51 (2)	1/28 (3.6)	0/29 (0)
		Moderate	0/50 (0)	0/51 (0)	0/28 (0)	0/29 (0)
		Severe	0/50 (0)	0/51 (0)	0/28 (0)	0/29 (0)
	1	Mild	0/50 (0)	1/52 (1.9)	0/28 (0)	2/29 (6.9)
		Moderate	0/50 (0)	0/52 (0)	0/28 (0)	0/29 (0)
		Severe	0/50 (0)	0/52 (0)	0/28 (0)	0/29 (0)
	2	Mild	0/50 (0)	0/52 (0)	0/28 (0)	1/29 (3.4)
		Moderate	0/50 (0)	1/52 (1.9)	0/28 (0)	0/29 (0)
		Severe	0/50 (0)	0/52 (0)	0/28 (0)	0/29 (0)
	3	Mild	0/50 (0)	1/52 (1.9)	0/28 (0)	0/29 (0)
		Moderate	1/50 (2)	0/52 (0)	0/28 (0)	0/29 (0)
		Severe	0/50 (0)	0/52 (0)	0/28 (0)	0/29 (0)
	4	Mild	1/50 (2)	1/51 (2)	0/28 (0)	0/29 (0)
		Moderate	0/50 (0)	0/51 (0)	0/28 (0)	0/29 (0)
		Severe	0/50 (0)	0/51 (0)	0/28 (0)	0/29 (0)
	5	Mild	0/49 (0)	1/51 (2)	0/28 (0)	1/29 (3.4)
		Moderate	1/49 (2)	0/51 (0)	0/28 (0)	0/29 (0)
		Severe	0/49 (0)	0/51 (0)	0/28 (0)	0/29 (0)
	6	Mild	0/50 (0)	1/50 (2)	0/28 (0)	0/29 (0)
		Moderate	0/50 (0)	0/50 (0)	0/28 (0)	0/29 (0)
		Severe	0/50 (0)	0/50 (0)	0/28 (0)	0/29 (0)
Sweating	0	Mild	0/50 (0)	5/52 (9.6)	1/28 (3.6)	0/29 (0)
		Moderate	1/50 (2)	1/52 (1.9)	0/28 (0)	0/29 (0)
		Severe	0/50 (0)	0/52 (0)	0/28 (0)	0/29 (0)
	1	Mild	2/50 (4)	4/52 (7.7)	0/28 (0)	2/29 (6.9)
		Moderate	1/50 (2)	1/52 (1.9)	0/28 (0)	0/29 (0)
		Severe	0/50 (0)	0/52 (0)	0/28 (0)	0/29 (0)
	2	Mild	2/50 (4)	2/52 (3.8)	0/28 (0)	1/29 (3.4)
		Moderate	0/50 (0)	1/52 (1.9)	0/28 (0)	0/29 (0)
		Severe	0/50 (0)	0/52 (0)	0/28 (0)	0/29 (0)
	3	Mild	0/50 (0)	2/52 (3.8)	0/28 (0)	1/29 (3.4)
		Moderate	0/50 (0)	0/52 (0)	0/28 (0)	0/29 (0)
		Severe	0/50 (0)	0/52 (0)	0/28 (0)	0/29 (0)
	4	Mild	0/50 (0)	3/51 (5.9)	0/28 (0)	1/29 (3.4)
		Moderate	1/50 (2)	0/51 (0)	0/28 (0)	0/29 (0)
		Severe	0/50 (0)	0/51 (0)	0/28 (0)	0/29 (0)
	5	Mild	0/50 (0)	3/51 (5.9)	0/28 (0)	1/29 (3.4)
		Moderate	0/50 (0)	0/51 (0)	0/28 (0)	0/29 (0)
		Severe	0/50 (0)	0/51 (0)	0/28 (0)	0/29 (0)

	6	Mild	0/50 (0)	1/50 (2)	0/28 (0)	0/29 (0)
		Moderate	0/50 (0)	1/50 (2)	0/28 (0)	0/29 (0)
		Severe	0/50 (0)	0/50 (0)	0/28 (0)	0/29 (0)
Swelling	0	Mild	1/49 (2)	0/51 (0)	0/27 (0)	1/29 (3.4)
		Moderate	0/49 (0)	0/51 (0)	0/27 (0)	0/29 (0)
		Severe	0/49 (0)	0/51 (0)	0/27 (0)	0/29 (0)
	1	Mild	0/49 (0)	0/51 (0)	0/27 (0)	1/29 (3.4)
		Moderate	0/49 (0)	0/51 (0)	0/27 (0)	0/29 (0)
		Severe	0/49 (0)	0/51 (0)	0/27 (0)	0/29 (0)
	2	Mild	0/49 (0)	0/52 (0)	0/27 (0)	1/29 (3.4)
		Moderate	0/49 (0)	0/52 (0)	0/27 (0)	0/29 (0)
		Severe	0/49 (0)	0/52 (0)	0/27 (0)	0/29 (0)
	3	Mild	0/49 (0)	0/52 (0)	0/27 (0)	0/29 (0)
		Moderate	0/49 (0)	0/52 (0)	0/27 (0)	0/29 (0)
		Severe	0/49 (0)	0/52 (0)	0/27 (0)	0/29 (0)
	4	Mild	0/49 (0)	0/51 (0)	0/27 (0)	0/29 (0)
		Moderate	0/49 (0)	0/51 (0)	0/27 (0)	0/29 (0)
		Severe	0/49 (0)	0/51 (0)	0/27 (0)	0/29 (0)
	5	Mild	0/48 (0)	0/51 (0)	0/26 (0)	0/29 (0)
		Moderate	0/48 (0)	0/51 (0)	0/26 (0)	0/29 (0)
		Severe	0/48 (0)	0/51 (0)	0/26 (0)	0/29 (0)
	6	Mild	0/47 (0)	0/51 (0)	0/26 (0)	0/29 (0)
		Moderate	0/47 (0)	0/51 (0)	0/26 (0)	0/29 (0)
		Severe	0/47 (0)	0/51 (0)	0/26 (0)	0/29 (0)
Temperature	0	Mild	0/48 (0)	0/52 (0)	0/27 (0)	0/29 (0)
		Moderate	0/48 (0)	0/52 (0)	0/27 (0)	0/29 (0)
		Severe	0/48 (0)	0/52 (0)	0/27 (0)	0/29 (0)
		Grade 4	0/48 (0)	0/52 (0)	0/27 (0)	0/29 (0)
	1	Mild	0/49 (0)	0/52 (0)	0/27 (0)	0/29 (0)
		Moderate	0/49 (0)	0/52 (0)	0/27 (0)	0/29 (0)
		Severe	0/49 (0)	0/52 (0)	0/27 (0)	0/29 (0)
		Grade 4	0/49 (0)	0/52 (0)	0/27 (0)	0/29 (0)
	2	Mild	0/49 (0)	0/51 (0)	0/27 (0)	0/29 (0)
		Moderate	0/49 (0)	0/51 (0)	1/27 (3.7)	0/29 (0)
		Severe	0/49 (0)	0/51 (0)	0/27 (0)	0/29 (0)
		Grade 4	0/49 (0)	0/51 (0)	0/27 (0)	0/29 (0)
	3	Mild	0/50 (0)	0/51 (0)	0/27 (0)	0/29 (0)
		Moderate	0/50 (0)	0/51 (0)	0/27 (0)	0/29 (0)
		Severe	0/50 (0)	0/51 (0)	0/27 (0)	0/29 (0)
		Grade 4	0/50 (0)	0/51 (0)	0/27 (0)	0/29 (0)
	4	Mild	0/49 (0)	0/50 (0)	0/27 (0)	0/29 (0)
		Moderate	0/49 (0)	0/50 (0)	0/27 (0)	0/29 (0)
		Severe	0/49 (0)	0/50 (0)	0/27 (0)	0/29 (0)
		Grade 4	0/49 (0)	0/50 (0)	0/27 (0)	0/29 (0)
	5	Mild	0/46 (0)	0/50 (0)	0/26 (0)	0/29 (0)
		Moderate	0/46 (0)	0/50 (0)	0/26 (0)	0/29 (0)
		Severe	0/46 (0)	0/50 (0)	0/26 (0)	0/29 (0)
		Grade 4	0/46 (0)	0/50 (0)	0/26 (0)	0/29 (0)
	6	Mild	0/44 (0)	1/49 (2)	0/24 (0)	0/29 (0)
		Moderate	0/44 (0)	0/49 (0)	0/24 (0)	0/29 (0)
		Severe	0/44 (0)	0/49 (0)	0/24 (0)	0/29 (0)
		Grade 4	0/44 (0)	0/49 (0)	0/24 (0)	0/29 (0)
Tenderness	0	Mild	2/50 (4)	6/52 (11.5)	0/28 (0)	3/29 (10.3)
		Moderate	0/50 (0)	2/52 (3.8)	0/28 (0)	1/29 (3.4)
		Severe	0/50 (0)	1/52 (1.9)	0/28 (0)	0/29 (0)
	1	Mild	2/50 (4)	6/52 (11.5)	0/28 (0)	1/29 (3.4)
		Moderate	0/50 (0)	2/52 (3.8)	0/28 (0)	1/29 (3.4)

		Severe	0/50 (0)	1/52 (1.9)	0/28 (0)	0/29 (0)
	2	Mild	1/50 (2)	3/52 (5.8)	0/28 (0)	2/29 (6.9)
		Moderate	0/50 (0)	2/52 (3.8)	0/28 (0)	0/29 (0)
		Severe	0/50 (0)	0/52 (0)	0/28 (0)	0/29 (0)
	3	Mild	0/49 (0)	3/52 (5.8)	0/28 (0)	1/29 (3.4)
		Moderate	1/49 (2)	0/52 (0)	0/28 (0)	0/29 (0)
		Severe	0/49 (0)	0/52 (0)	0/28 (0)	0/29 (0)
	4	Mild	0/50 (0)	1/51 (2)	0/28 (0)	1/29 (3.4)
		Moderate	0/50 (0)	0/51 (0)	0/28 (0)	0/29 (0)
		Severe	0/50 (0)	0/51 (0)	0/28 (0)	0/29 (0)
	5	Mild	0/50 (0)	1/51 (2)	0/27 (0)	0/29 (0)
		Moderate	0/50 (0)	0/51 (0)	0/27 (0)	0/29 (0)
		Severe	0/50 (0)	0/51 (0)	0/27 (0)	0/29 (0)
	6	Mild	0/50 (0)	0/51 (0)	0/27 (0)	0/29 (0)
		Moderate	0/50 (0)	0/51 (0)	0/27 (0)	0/29 (0)
		Severe	0/50 (0)	0/51 (0)	0/27 (0)	0/29 (0)
Weakness	0	Mild	5/50 (10)	6/52 (11.5)	0/28 (0)	2/29 (6.9)
		Moderate	1/50 (2)	1/52 (1.9)	1/28 (3.6)	0/29 (0)
		Severe	0/50 (0)	0/52 (0)	0/28 (0)	0/29 (0)
	1	Mild	5/50 (10)	4/52 (7.7)	0/28 (0)	1/29 (3.4)
		Moderate	1/50 (2)	0/52 (0)	1/28 (3.6)	0/29 (0)
		Severe	0/50 (0)	1/52 (1.9)	0/28 (0)	0/29 (0)
	2	Mild	4/50 (8)	3/52 (5.8)	0/28 (0)	3/29 (10.3)
		Moderate	0/50 (0)	2/52 (3.8)	0/28 (0)	0/29 (0)
		Severe	0/50 (0)	0/52 (0)	0/28 (0)	0/29 (0)
	3	Mild	1/50 (2)	1/52 (1.9)	0/28 (0)	1/29 (3.4)
		Moderate	0/50 (0)	0/52 (0)	0/28 (0)	0/29 (0)
		Severe	0/50 (0)	0/52 (0)	0/28 (0)	0/29 (0)
	4	Mild	2/50 (4)	0/51 (0)	0/28 (0)	0/29 (0)
		Moderate	0/50 (0)	0/51 (0)	0/28 (0)	1/29 (3.4)
		Severe	0/50 (0)	0/51 (0)	0/28 (0)	0/29 (0)
	5	Mild	2/50 (4)	1/51 (2)	2/28 (7.1)	0/29 (0)
		Moderate	0/50 (0)	0/51 (0)	0/28 (0)	0/29 (0)
		Severe	0/50 (0)	0/51 (0)	0/28 (0)	0/29 (0)
	6	Mild	2/50 (4)	0/50 (0)	0/28 (0)	2/29 (6.9)
		Moderate	0/50 (0)	1/50 (2)	0/28 (0)	0/29 (0)
		Severe	0/50 (0)	0/50 (0)	0/28 (0)	0/29 (0)

AE=adverse event; HIV=human immunodeficiency virus; PLWH=people living with HIV; SOB, shortness of breath.

Supplementary Table 5. AEs reported by HIV-negative participants stratified by treatment group.

	Number of events (incidence risk per participant)		
	Overall (N=58)	Placebo (n=29)	Vaccine (n=29)
AE	237 (401·7)	142 (489·7)	95 (327·6)
**General disorders and administration site conditions**	**78 (134·5)**	**46 (158·6)**	**32 (110·3)**
General system disorders NEC	77 (132·8)	46 (158·6)	31 (106·9)
Body temperature conditions	1 (1·7)	0 (<0·1)	1 (3·4)
**Injury, poisoning and procedural complications**	**1 (1·7)**	**1 (3·4)**	**0 (<0·1)**
Injuries NEC	1 (1·7)	1 (3·4)	0 (<0·1)
**Investigations**	**1 (1·7)**	**1 (3·4)**	**0 (<0·1)**
Microbiology and serology investigations	1 (1·7)	1 (3·4)	0 (<0·1)
**Reproductive system and breast disorders**	**1 (1·7)**	**0 (<0·1)**	**1 (3·4)**
Vulvovaginal disorders (excl infections and inflammations)	1 (1·7)	0 (<0·1)	1 (3·4)
**Vascular disorders**	**1 (1·7)**	**0 (<0·1)**	**1 (3·4)**
Vascular hypertensive disorders	1 (1·7)	0 (<0·1)	1 (3·4)
**Nervous system disorders**	**43 (74·1)**	**28 (96·6)**	**15 (51·7)**
Headaches	35 (60·3)	23 (79·3)	12 (41·4)
Cranial nerve disorders (excl neoplasms)	5 (8·6)	3 (10·3)	2 (6·9)
Neurological disorders NEC	3 (5·2)	2 (6·9)	1 (3·4)
**Gastrointestinal disorders**	**33 (56·9)**	**18 (62·1)**	**15 (51·7)**
Gastrointestinal signs and symptoms	18 (31)	9 (31)	9 (31)
Gastrointestinal motility and defaecation conditions	13 (22·4)	8 (27·6)	5 (17·2)
Gastrointestinal vascular conditions	1 (1·7)	1 (3·4)	0 (<0·1)
Oral soft tissue conditions	1 (1·7)	0 (<0·1)	1 (3·4)
**Infections and infestations**	**27 (46·6)**	**18 (62·1)**	**9 (31)**
Infections - pathogen unspecified	23 (39·7)	16 (55·2)	7 (24·1)
Bacterial infectious disorders	1 (1·7)	1 (3·4)	0 (<0·1)
Fungal infectious disorders	1 (1·7)	1 (3·4)	0 (<0·1)
Mycobacterial infectious disorders	1 (1·7)	0 (<0·1)	1 (3·4)
Viral infectious disorders	1 (1·7)	0 (<0·1)	1 (3·4)
**Respiratory, thoracic, and mediastinal disorders**	**27 (46·6)**	**19 (65·5)**	**8 (27·6)**
Respiratory disorders NEC	13 (22·4)	9 (31)	4 (13·8)
Respiratory tract signs and symptoms	7 (12·1)	4 (13·8)	3 (10·3)
Upper respiratory tract disorders (excl infections)	7 (12·1)	6 (20·7)	1 (3·4)
**Musculoskeletal and connective tissue disorders**	**17 (29·3)**	**8 (27·6)**	**9 (31)**
Muscle disorders	9 (15·5)	4 (13·8)	5 (17·2)
Joint disorders	5 (8·6)	3 (10·3)	2 (6·9)
Musculoskeletal and connective tissue disorders NEC	3 (5·2)	1 (3·4)	2 (6·9)
**Skin and subcutaneous tissue disorders**	**6 (10·3)**	**2 (6·9)**	**4 (13·8)**
Epidermal and dermal conditions	6 (10·3)	2 (6·9)	4 (13·8)
**Ear and labyrinth disorders**	**1 (1·7)**	**0 (<0·1)**	**1 (3·4)**
External ear disorders (excl congenital)	1 (1·7)	0 (<0·1)	1 (3·4)
**Immune system disorders**	**1 (1·7)**	**1 (3·4)**	**0 (<0·1)**

Allergic conditions	1 (1·7)	1 (3·4)	0 (<0·1)

Incidence risk calculation is based on 58 trial participants (29 placebo and 29 vaccine). This excludes 13 participants who were SARS-CoV-2 seropositive at baseline.
AE=adverse event; HIV=human immunodeficiency virus; NEC=not elsewhere classified; SARS-CoV-2=severe acute respiratory syndrome coronavirus 2.

Supplementary Table 6. AEs reported by PLWH, stratified by treatment group.

	Number of events (Incidence risk per participant)		
	**Overall (N=103)**	**Placebo (n=51)**	**Vaccine (n=52)**
**AE**	**164 (159·2)**	**78 (152·9)**	**86 (165·4)**
**General disorders and administration site conditions**	**44 (42·7)**	**22 (43·1)**	**22 (42·3)**
General system disorders NEC	41 (39·8)	20 (39·2)	21 (40·4)
Administration site reactions	3 (2·9)	2 (3·9)	1 (1·9)
**Blood and lymphatic system disorders**	**2 (1·9)**	**0 (<0·1)**	**2 (3·8)**
Anaemias nonhaemolytic and marrow depression	2 (1·9)	0 (<0·1)	2 (3·8)
**Ear and labyrinth disorders**	**2 (1·9)**	**0 (<0·1)**	**2 (3·8)**
Aural disorders NEC	2 (1·9)	0 (<0·1)	2 (3·8)
**Eye disorders**	**2 (1·9)**	**0 (<0·1)**	**2 (3·8)**
Eye disorders NEC	1 (1)	0 (<0·1)	1 (1·9)
Ocular infections, irritations, and inflammations	1 (1)	0 (<0·1)	1 (1.9)
**Injury, poisoning, and procedural complications**	**2 (1·9)**	**2 (3·9)**	**0 (<0·1)**
Injuries NEC	2 (1·9)	2 (3·9)	0 (<0·1)
**Vascular disorders**	**2 (1·9)**	**0 (<0.1)**	**2 (3·8)**
Vascular hypertensive disorders	2 (1·9)	0 (<0·1)	2 (3·8)
**Skin and subcutaneous tissue disorders**	**1 (1)**	**0 (<0·1)**	**1 (1·9)**
Skin appendage conditions	1 (1)	0 (<0·1)	1 (1·9)
**Nervous system disorders**	**26 (25·2)**	**10 (19·6)**	**16 (30·8)**
Headaches	22 (21·4)	9 (17·6)	13 (25)
Neurological disorders NEC	4 (3·9)	1 (2)	3 (5·8)
**Infections and infestations**	**25 (24·3)**	**12 (23·5)**	**13 (25)**
Infections - pathogen unspecified	22 (21·4)	11 (21·6)	11 (21·2)
Viral infectious disorders	2 (1·9)	1 (2)	1 (1·9)
Bacterial infectious disorders	1 (1)	0 (<0·1)	1 (1·9)
**Gastrointestinal disorders**	**19 (18·4)**	**8 (15·7)**	**11 (21·2)**
Gastrointestinal motility and defaecation conditions	14 (13·6)	6 (11·8)	8 (15·4)
Gastrointestinal signs and symptoms	3 (2·9)	1 (2)	2 (3·8)
Dental and gingival conditions	1 (1)	1 (2)	0 (<0.1)
Gastrointestinal vascular conditions	1 (1)	0 (<0.1)	1 (1.9)
**Musculoskeletal and connective tissue disorders**	**13 (12·6)**	**8 (15·7)**	**5 (9·6)**
Musculoskeletal and connective tissue disorders NEC	5 (4·9)	4 (7·8)	1 (1·9)
Joint disorders	4 (3·9)	1 (2)	3 (5·8)
Muscle disorders	4 (3·9)	3 (5·9)	1 (1·9)
**Investigations**	**10 (9·7)**	**9 (17·6)**	**1 (1·9)**
Water, electrolyte, and mineral investigations	5 (4·9)	4 (7·8)	1 (1·9)
Hepatobiliary investigations	4 (3·9)	4 (7·8)	0 (<0·1)
Enzyme investigations NEC	1 (1)	1 (2)	0 (<0·1)
**Metabolism and nutrition disorders**	8 (7·8)	4 (7·8)	4 (7·7)
Electrolyte and fluid balance conditions	8 (7·8)	4 (7·8)	4 (7·7)
**Respiratory, thoracic, and mediastinal disorders**	5 (4·9)	2 (3·9)	3 (5·8)

Respiratory disorders NEC	4 (3·9)	2 (3·9)	2 (3·8)
Respiratory tract signs and symptoms	1 (1)	0 (<0·1)	1 (1·9)
**Reproductive system and breast disorders**	3 (2·9)	1 (2)	2 (3·8)
Menstrual cycle and uterine bleeding disorders	1 (1)	1 (2)	0 (<0·1)
Reproductive tract disorders NEC	1 (1)	0 (<0·1)	1 (1·9)
Uterine, pelvic, and broad ligament disorders	1 (1)	0 (<0·1)	1 (1·9)

Incidence risk calculation is based on 103 trial participants (51 placebo and 52 vaccine). This excludes 13 participants who were SARS-CoV-2 seropositive at baseline.
AE=adverse event; HIV=human immunodeficiency virus; NEC=not elsewhere classified; PLWH=people living with HIV; SARS-CoV-2=severe acute respiratory syndrome coronavirus 2.

Supplementary Table 7. SAEs reported by HIV-negative participants and PLWH.

Group	Treatment	Classification	Relatedness	Diagnosis
HIV-negative participants	Vaccine	Important medical event	Definite	Severe fever (40·5°C, resolved with paracetamol)
		Hospitalisation/prolongation	Not related	Right angle of mandible fracture
		Important medical event	Not related	Pulmonary tuberculosis
		Hospitalisation/prolongation	Not related	Chest pain
	Placebo	Death	Not related	Suicide
		Hospitalisation/prolongation	Not related	Left axillary abscess
PLWH	Placebo	Important medical event	Unlikely	Severely elevated ALT

ALT=alanine aminotransferase; HIV=human immunodeficiency virus; PLWH=people living with HIV; SAE=serious adverse event.

Supplementary Table 8. Number of participants with a positive SARS-CoV-2 PCR test, stratified by HIV status and time since vaccine dose.

	**HIV-negative participants (n=70)**				**PLWH (n=104)**			
	**Time from vaccine dose, days**							
	**≤0**	**>0 to ≤28**	**>28 to ≤42**	**>42**	**≤0**	**>0 to ≤28**	**>28 to ≤42**	**>42**
PCR− throughout trial	46				96			
**Symptom severity in PCR+**								
Asymptomatic	4 (3P/1V)	2 (2V)	0	0	0	0	1 (1V)	0
Mild	3 (1P/2V)	3 (2P/1V)	0	2 (2P)	1 (1P)	2 (1P/1V)	1 (1P)	1 (1V)
Moderate	0	-	0	0	0	0	1 (1V)	0
Other (displayed some symptoms, but did not meet protocol definition of mild COVID-19 disease)	5 (3P/2V)	4 (3P/1V)	0	1 (1P)	0	0	0	1 (1V)

COVID-19=coronavirus disease 2019; HIV=human immunodeficiency virus; P=placebo; PCR=polymerase chain reaction; PLWH=people living with HIV; SARS-CoV-2=severe acute respiratory syndrome coronavirus 2; V=vaccine.

Supplementary Table 9. FLS IgG responses in participants who were SARS-CoV-2 seronegative at baseline, stratified by HIV status.

Arm	Measure	Day 0 (baseline)		p-value†
		**HIV-negative participants**	**PLWH**	
Placebo	GMC (95% CI), BAU/mL	1·5 (0·9–2·6); n=26	1·2 (0·7–2·0); n=33	0.46
	% seropositive	3·8 (0·7–18·9); 1/26	0 (0–10·4); 0/33	0.44
Vaccine	GMC (95% CI), BAU/mL	1·3 (0·9–1·9); n=27	1·34 (0·8–2·3); n=37	0.93
	% seropositive	0 (0–12·5); 0/27	8·1 (2·8–21·3); 3/37	0.26
		**Day 28 (booster dose)**		
		**HIV-negative participants**	**PLWH**	
Placebo	GMC (95% CI), BAU/mL	1·5 (0·7–3·6); n=20	2·1 (1·1–4·2); n=33	0.56
	% seropositive	10 (2·8–30·1); 2/20	9·1 (3·1–23·6); 3/33	>0.99
	% seroresponsive*	0 (0–79·3); 0/1	–	–
Vaccine	GMC (95% CI), BAU/mL	112·3 (61·7–204·4); n=23	163·7 (89·9–298·1); n=36	0.37
	% seropositive	78·3 (58·1–90·3); 18/23	86·1 (71·3–93·9); 31/36	0.49
	% seroresponsive	–	100 (43·9–100); 3/3	–
		**Day 42 (14 days post booster)**		
		**HIV-negative participants**	**PLWH**	
Placebo	GMC (95% CI), BAU/mL	1·5 (0·7–3·3); n=21	1·91 (0·9–4·1); n=30	0.68
	% seropositive	9·5 (2·7–28·9); 2/21	6·7 (1·8–21·3); 2/30	>0.99
	% seroresponsive	0 (0–65·8); 0/2	0 (0–56·1); 0/3	>0.99
Vaccine	GMC (95% CI), BAU/mL	504·9 (337·1–756·2); n=23	453·1 (267·4–767·7); n=32	0.74
	% seropositive	95·7 (79–99·2); 22/23	93·8 (79·9-98·3); 30/32	>0.99
	% seroresponsive	72·2 (49·1–87·5); 13/18	63·0 (44·2–78·5); 17/27	0.75

*Samples considered seroresponsive if ≥2-fold increase. †p-value was calculated using Fisher's exact test.
BAU=binding antibody unit; CI=confidence interval; FLS=full-length spike; GMC=geometric mean concentration; HIV=human immunodeficiency virus; IgG=immunoglobulin G; PLWH=people living with HIV; SARS-CoV-2=severe acute respiratory syndrome coronavirus 2.

Supplementary Table 10. RBD IgG response in participants who were SARS-CoV-2 seronegative at baseline, stratified by HIV status.

Arm	Measure	Day 0 (baseline)		p-value†
		**HIV-negative participants**	**PLWH**	
Placebo	GMC (95% CI), BAU/mL	1·0 (0·5–1·9); n=26	0·5 (0·3–0·9); n=33	0.12
Vaccine	GMC (95% CI), BAU/mL	1·2 (0·9–1·7); n=27	0·8 (0·5–1·3); n=37	0.14
		**Day 28 (booster dose)**		
		**HIV-negative participants**	**PLWH**	
Placebo	GMC (95% CI), BAU/mL	1·2 (0·5–2·9); n=20	1·0 (0·5–2·1); n=33	0.85
	% seropositive	10 (2·8–30·1); 2/20	9·1 (3·1–23·6); 3/33	>0.99
Vaccine	GMC (95% CI), BAU/mL	51·1 (24·3–107·4); n=23	80·0 (40·3–158·8); n=36	0.37
	% seropositive	65·2 (44·9–81·2); 15/23	69·4 (53·1–82); 25/36	0.78
		**Day 42 (14 days post booster)**		
		**HIV-negative participants**	**PLWH**	
Placebo	GMC (95% CI), BAU/mL	1·1 (0·5–2·8); n=21	0·9 (0·4–2·1); n=30	0.74
	% seropositive	9·5 (2·7–28·9); 2/21	6·7 (1·8–21·3); 2/30	>0.99
	% seroresponsive*	0 (0–65·8); 0/2	0 (0–56·1); 0/3	>0.99
Vaccine	GMC (95% CI), BAU/mL	364·2 (238·6–555·8); n=23	347·8 (195·0–620·4); n=32	0.90
	% seropositive	95·7 (79–99·2); 22/23	87·5 (71·9–95); 28/32	0.39
	% seroresponsive	66·7 (41·7–84·8); 10/15	68·2 (47·3–83·6); 15/22	>0.99

*Samples considered seroresponsive if ≥2-fold increase. †p-value was calculated using Fisher's exact test.
BAU=binding antibody unit; CI=confidence interval; GMC=geometric mean concentration; HIV=human immunodeficiency virus; IgG=immunoglobulin G; PLWH=people living with HIV; RBD=receptor-binding domain; SARS-CoV-2=severe acute respiratory syndrome coronavirus 2.

Supplementary Table 11. FLS IgG responses in PLWH, stratified by SARS-CoV-2 serostatus at baseline.

Arm	Measure	Day 0 (baseline)		p-value[†]
		**SARS-CoV-2 seronegative**	**SARS-CoV-2 seropositive**	
Placebo	GMC (95% CI), BAU/mL	1·2 (0·7–2·0); n=33	194·5 (130·1–290·9); n=17	<0.001
	% seropositive	0 (0–10·4); 0/33	100 (81·6–100); 17/17	<0.001
	% seroresponsive[*]	–	–	–
Vaccine	GMC (95% CI), BAU/mL	1·3 (0·8–2·3); n=37	208·8 (111·9–389·8); n=15	<0.001
	% seropositive	8·1 (2·8–21·3); 3/37	100 (79·6–100); 15/15	<0.001
	% seroresponsive	–	–	–
		**Day 28 (booster dose)**		
		**SARS-CoV-2 seronegative**	**SARS-CoV-2 seropositive**	
Placebo	GMC (95% CI), BAU/mL	2·1 (1·1–4·2); n=33	220·6 (137·2–354·8); n=16	<0.001
	% seropositive	9·1 (3·1–23·6); 3/33	100 (80·6–100); 16/16	<0.001
	% seroresponsive	–	6·2 (1·1–28·3); 1/16	–
Vaccine	GMC (95% CI), BAU/mL	163·7 (89·9–298·1); n=36	2942.2 (1803.1–4801.1); n=15	<0.001
	% seropositive	86·1 (71·3–93·9); 31/36	100 (79·6–100); 15/15	0.31
	% seroresponsive	100 (43·9–100); 3/3	86·7 (62·1–96·3); 13/15	>0.99
		**Day 42 (14 days post booster)**		
		**SARS-CoV-2 seronegative**	**SARS-CoV-2 seropositive**	
Placebo	GMC (95% CI), BAU/mL	1·9 (0·9–4·1); n=30	211·8 (129·7–345·9); n=16	<0.001
	% seropositive	6·7 (1·8–21·3); 2/30	100 (80·6–100); 16/16	<0.001
	% seroresponsive	0 (0–56·1); 0/3	0 (0–19·4); 0/16	>0.99
Vaccine	GMC (95% CI), BAU/mL	453·1 (267·4–767·7); n=32	3782.34 (2229.1–6417.9); n=15	<0.001
	% seropositive	93·8 (79·9–98·3); 30/32	100 (79·6–100); 15/15	>0.99
	% seroresponsive	63·0 (44·2–78·5); 17/27	20·0 (7·0–45·2); 3/15	0.01

[*]Samples considered seroresponsive if $\geq$2-fold increase. [†]p-value was calculated using Fisher's exact test.
BAU=binding antibody unit; CI=confidence interval; FLS=full-length spike GMC=geometric mean concentration; HIV=human immunodeficiency virus; IgG=immunoglobulin G; PLWH=people living with HIV; SARS-CoV-2=severe acute respiratory syndrome coronavirus 2.

Supplementary Table 12. RBD IgG responses in PLWH, stratified by SARS-CoV-2 serostatus at baseline.

Arm	Measure	Day 0 (baseline)		p-value†
		**SARS-CoV-2 seronegative**	**SARS-CoV-2 seropositive**	
Placebo	GMC (95% CI), BAU/mL	0·5 (0·3–0·9); n=33	162·3 (95·5–275·9); n=17	<0.001
	% seropositive	0 (0–10·4); 0/33	100 (81·6–100); 17/17	<0.001
Vaccine	GMC, (95% CI), BAU/mL	0·8 (0·5–1·3); n=37	136·4 (77·1–241·4); n=15	<0.001
	% seropositive	0 (0–9·4); 0/37	100 (79·6–100); 15/15	<0.001
		**Day 28 (booster dose)**		
		**SARS-CoV-2 seronegative**	**SARS-CoV-2 seropositive**	
Placebo	GMC (95% CI), BAU/mL	1·0 (0·5–2·1); n=33	188·4 (112·5–315·6); n=16	<0.001
	% seropositive	9·1 (3·1–23·6); 3/33	100 (80·6–100); 16/16	<0.001
	% seroresponsive*	–	6·2 (1·1–28·3); 1/16	–
Vaccine	GMC (95% CI), BAU/mL	80·0 (40·3–158·8); n=36	2378·7 (1237·9–4571·0); n=15	<0.001
	% seropositive	69·4 (53·1–82·0); 25/36	100 (79·6–100); 15/15	0.02
	% seroresponsive	–	93·3 (70·2–98·8); 14/15	–
		**Day 42 (14 days post booster)**		
		**SARS-CoV-2 seronegative**	**SARS-CoV-2 seropositive**	
Placebo	GMC (95% CI), BAU/mL	0·9 (0·4–2·1); n=30	166·8 (94·0–296·2); n=16	<0.001
	% seropositive	6·7 (1·8–21·3); 2/30	93·8 (71·7–98·9); 15/16	<0.001
	% seroresponsive	0 (0–56·1); 0/3	0 (0–19·4); 0/16	>0.99
Vaccine	GMC (95% CI), BAU/mL	347·8 (195·0–620·4); n=32	3183·2 (1628·6–6221·7); n=15	<0.001
	% seropositive	87·5 (71·9–95·0); 28/32	100 (79·6–100); 15/15	0.29
	% seroresponsive	68·2 (47·3–83·6); 15/22	40.0 (19·8–64·3); 6/15	0.11

*Samples considered seroresponsive if ≥2-fold increase. †p-value was calculated using Fisher's exact test.
BAU=binding antibody unit; CI=confidence interval; GMC=geometric mean concentration; HIV=human immunodeficiency virus; IgG=immunoglobulin G; PLWH=people living with HIV; RBD=receptor-binding domain; SARS-CoV-2=severe acute respiratory syndrome coronavirus 2.

Supplementary Table 13. Neutralising antibody responses in HIV-negative participants who were SARS-CoV-2 seronegative at baseline.

Arm	Measure	Day 0 (baseline)
Vaccine	GMT ID_{50}, (95% CI)	31·2 (1·9–526·6); n=2
	% seropositive	8 (2·2–25); 2/25
	% seroresponsive*	–
	**Measure**	**Day 28 (booster dose)**
	GMT ID_{50}, (95% CI)	135 (54·5–334·2); n=13
	% seropositive	59·1 (38·7–76·7); 13/22
	% seroresponsive	0 (0–65·8); 0/2
	**Measure**	**Day 42 (14 days post booster)**
	GMT ID_{50}, (95% CI)	316·4 (184·8–541·8); n=20
	% seropositive	95·2 (77·3–99·2); 20/21
	% seroresponsive	50 (25·4–74·6); 6/12

GMT are only described for RBD-seropositive participants. Twenty-six samples were analysed and one participant's result were unattainable at each time point due to background activity.

*Samples considered seroresponsive if $\geq$2-fold increase.

CI=confidence interval; GMT=geometric mean titre; HIV=human immunodeficiency virus; ID_{50}=inhibitory dilution (50%); RBD= receptor-binding domain; SARS-CoV-2=severe acute respiratory syndrome coronavirus 2.

Figures

Supplementary Figure 1. Correlation between MLV and pNL4 backbone-based neutralisation assays.

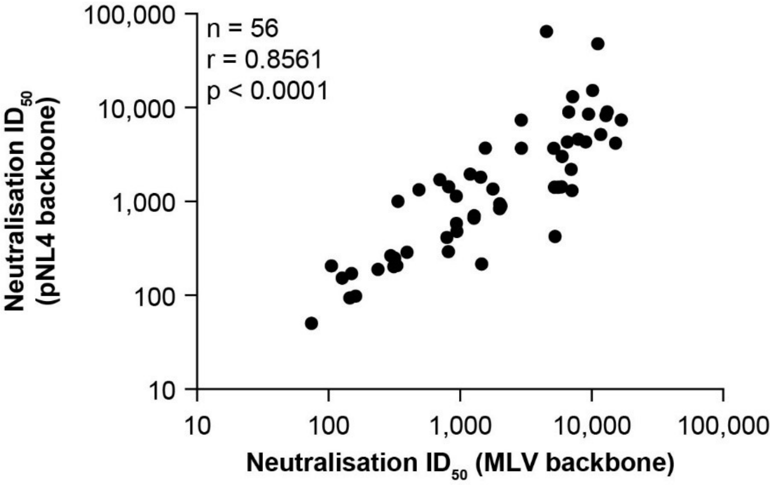

The SARS-CoV-2 spike was used to generate pseudotyped particles in two different lentiviral backbones, pNL4 or MLV, and ID_{50} titres were compared directly. The figure demonstrates high levels of concordance for 56 samples tested in the two different neutralisation assays.
HIV=human immunodeficiency virus; ID_{50}=inhibitory dilution (50%); MLV=murine leukaemia virus; pNL4=HIV vector pNL4; SARS-CoV-2=severe acute respiratory syndrome coronavirus 2.

Supplementary Figure 2. Correlation of plasma- and IgG-mediated neutralisation of Wuhan-1 (D614G WT).

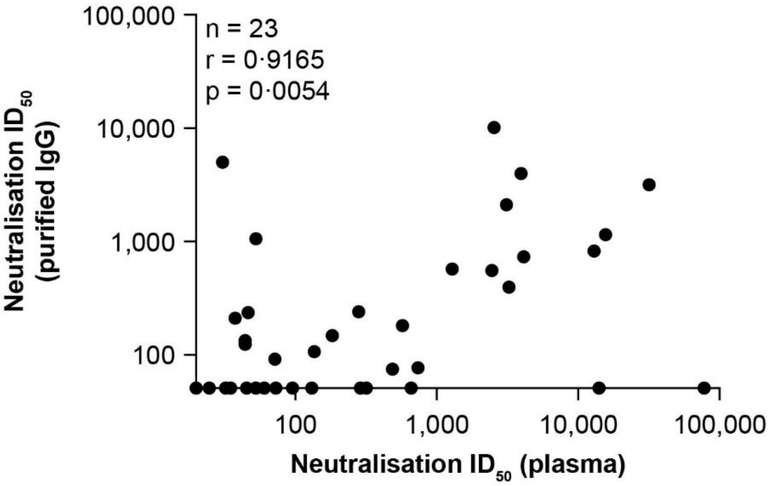

ID_{50}=inhibitory dilution (50%); IgG=immunoglobulin; WT=wild type

Supplementary Figure 3. Laboratory biochemistry abnormalities in a) HIV-negative participants who received the vaccine b) PLWH who received the vaccine c) HIV-negative participants who received placebo and d) PLWH who received placebo.

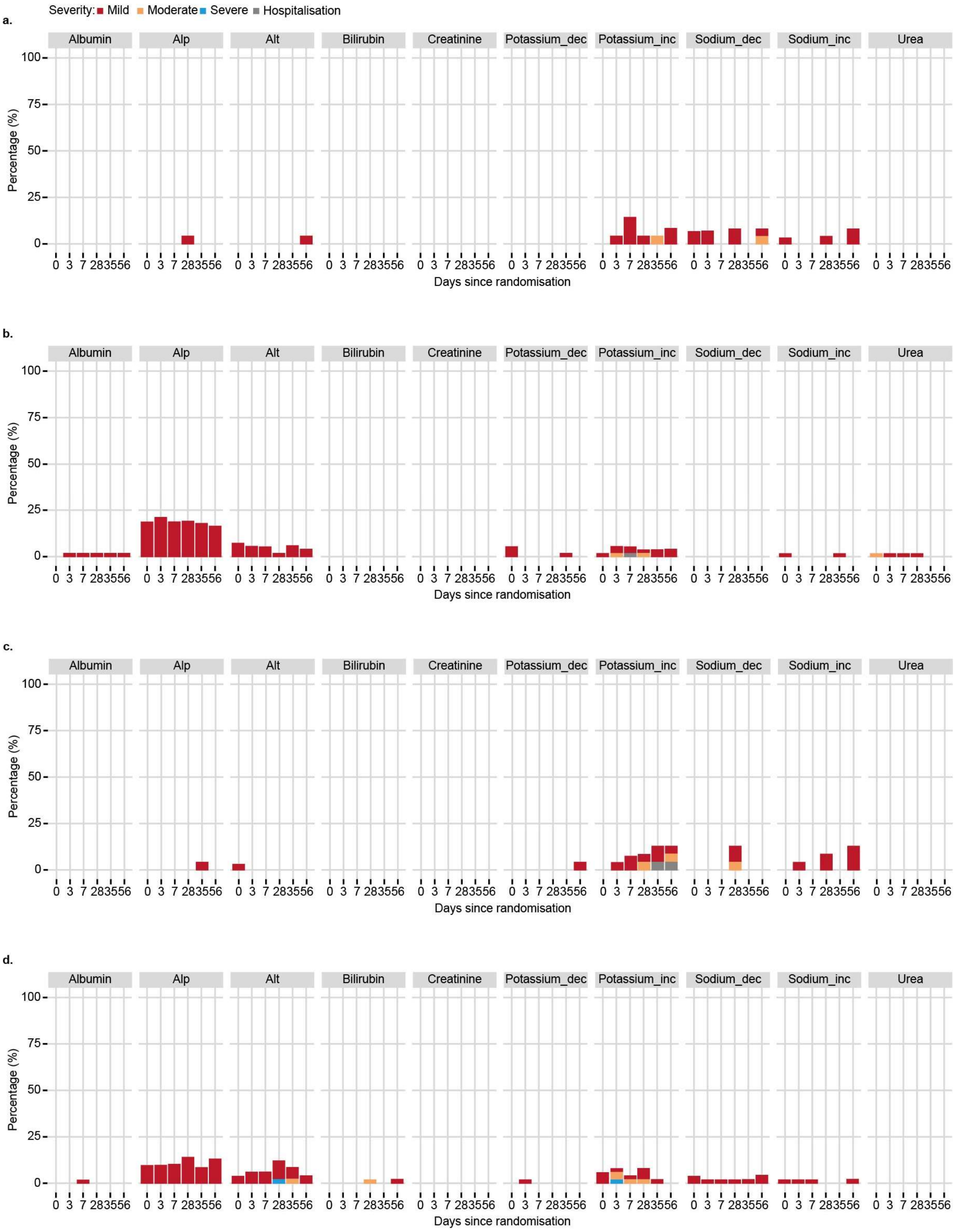

Alp=alkaline phosphatase; Alt, alanine aminotransferase; dec=decrease; HIV=human immunodeficiency virus; inc=increase; PLWH=people living with HIV.

Supplementary Figure 4. Laboratory haematology abnormalities in a) HIV-negative participants who received the vaccine b) PLWH who received the vaccine c) HIV-negative participants who received placebo and d) PLWH who received placebo.

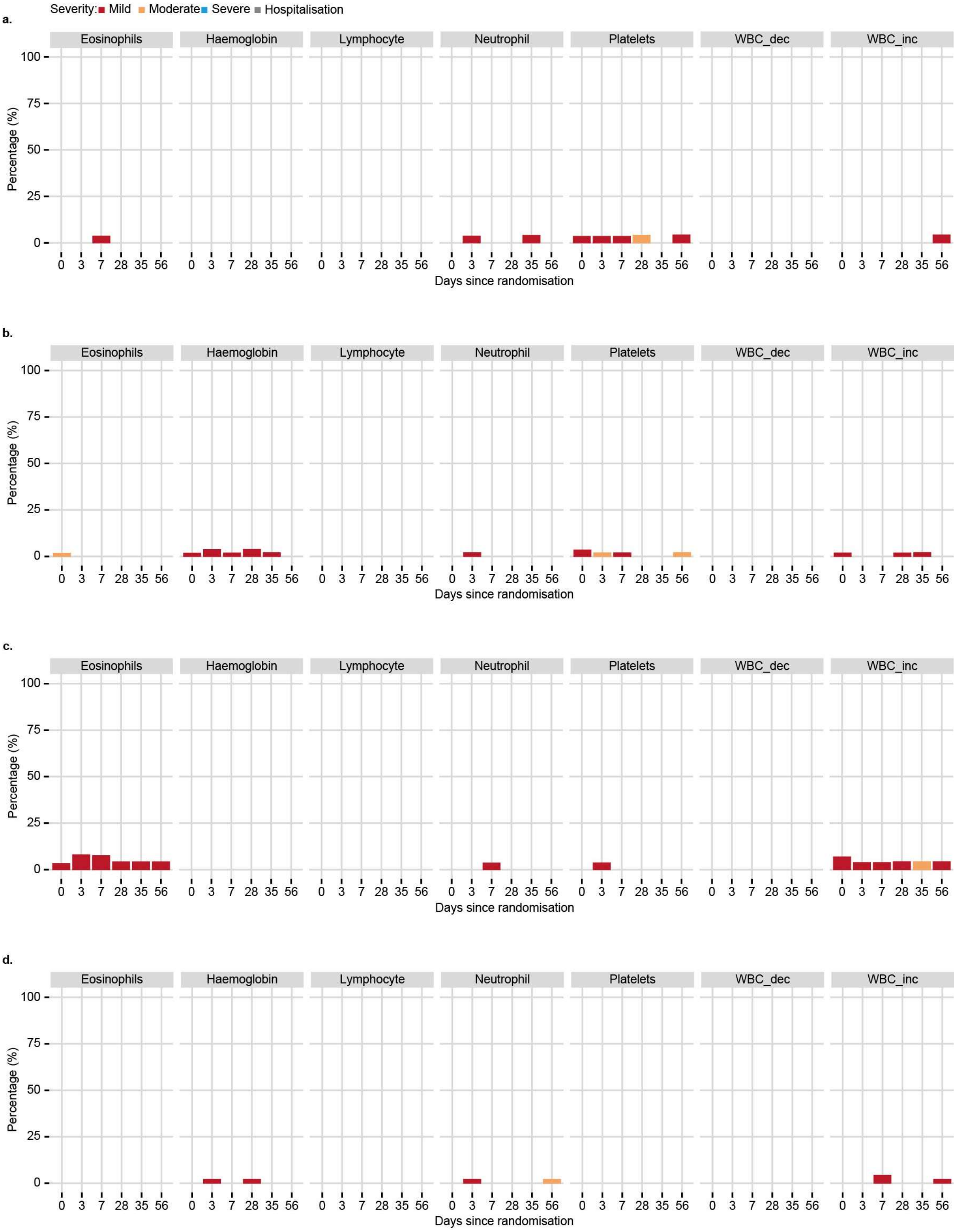

dec=decrease; HIV=human immunodeficiency virus; inc=increase PLWH=people living with HIV; WBC=white blood cell.

Supplementary Figure 5. Plasma IgG antibody binding to Wuhan-1 D614G WT and B·1·351 variant FLS stratified by day and HIV status in placebo recipients.

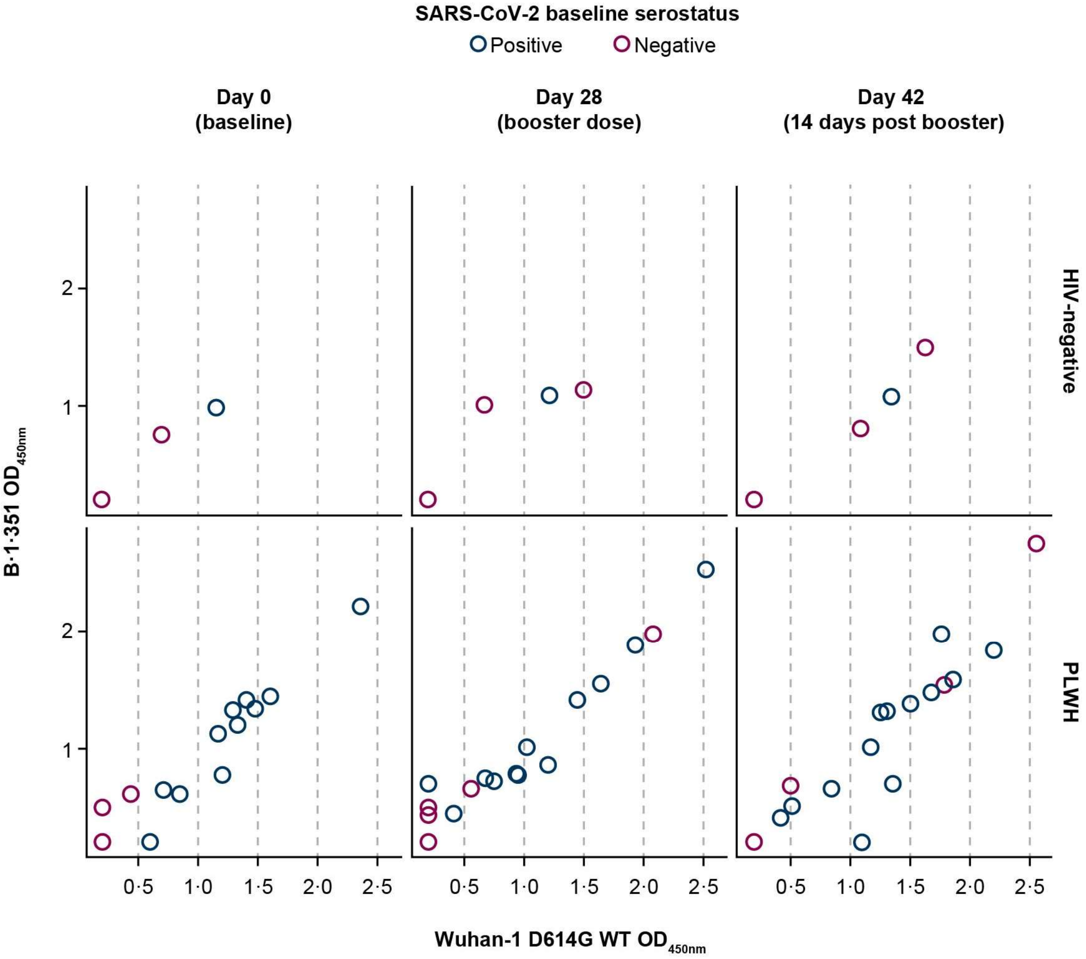

Data are plotted as area under the curve.

FLS=full-length spike; HIV=human immunodeficiency virus; IgG=immunoglobulin G; OD=optical density; PLWH=people living with HIV; SARS-CoV-2=severe acute respiratory syndrome coronavirus 2; WT=wild type.

Supplementary Figure 6. Correlation of neutralising antibody binding to Wuhan-1 D614G WT vs B·1·351 in vaccinees, stratified by HIV status.

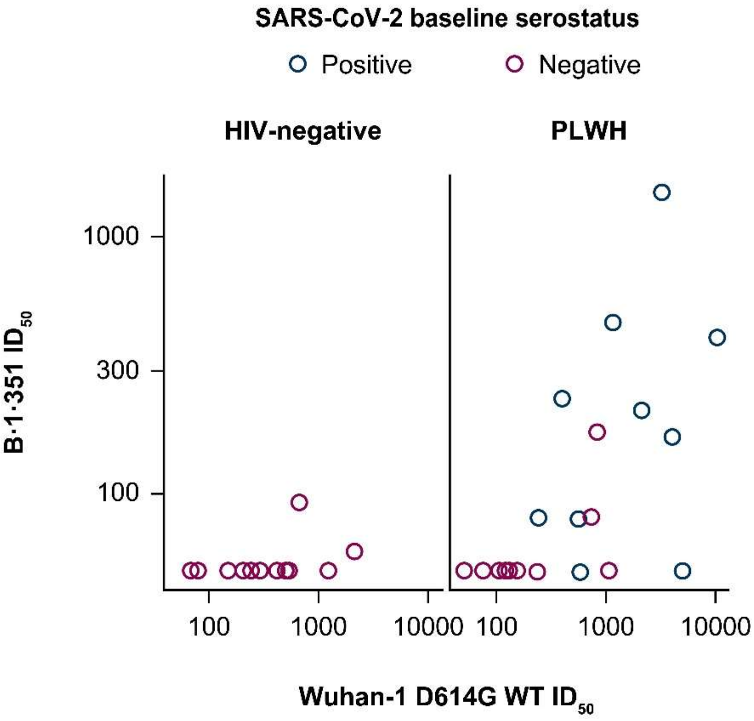

HIV=human immunodeficiency virus; ID_{50}=inhibitory dilution (50%); PLWH=people living with HIV; SARS-CoV-2=severe acute respiratory syndrome coronavirus 2; WT=wild type.

Supplementary Figure 7. Neutralisation activity against Wuhan-1 D614G WT and B·1·351 in PLWH who received placebo

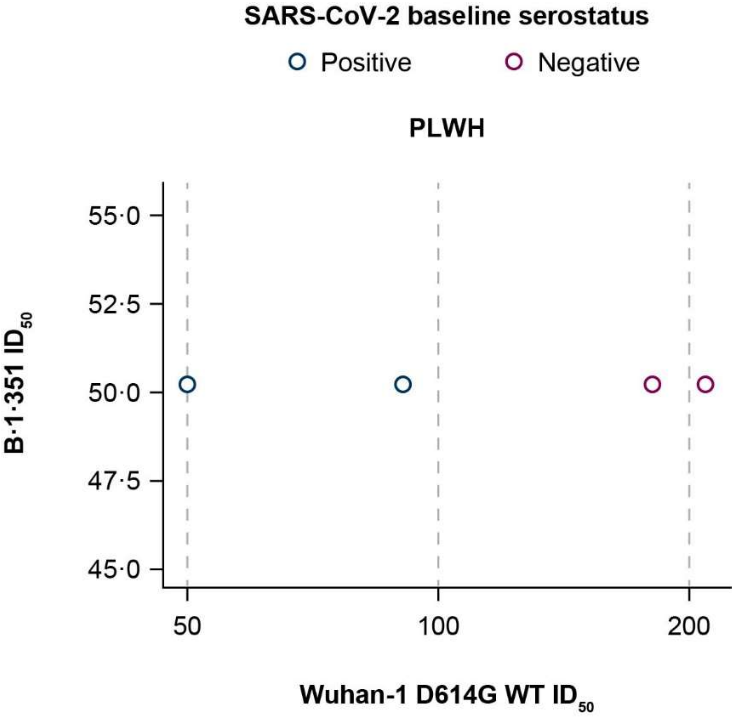

HIV=human immunodeficiency virus; ID_{50}=inhibitory dilution (50%); PLWH=people living with HIV; SARS-CoV-2=severe acute respiratory syndrome coronavirus 2; WT=wild type
